# Baseline gut microbiome and metabolome profiles predict weight loss after a structured lifestyle intervention

**DOI:** 10.1186/s40168-026-02501-x

**Published:** 2026-08-28

**Authors:** Benjamin Seethaler, Maryam Basrai, Nathalie M. Delzenne, Jens Walter, Nguyen K. Nguyen, Stephan C. Bischoff

**Affiliations:** 1 Institute of Nutritional Medicine, University of Hohenheim, Fruwirthstr. 12, Stuttgart, 70593 Germany; 2 Metabolism and Nutrition Research Group, Louvain Drug Research Institute (LDRI), UCLouvain, Université Catholique de Louvain, Brussels, Belgium; 3 APC Microbiome Ireland, Department of Medicine, and School of Microbiology, University College Cork, Cork, Ireland; 4 Department of Microbiology and Immunology, School of Biomedical Science, Faculty of Medicine and Health Sciences, McGill University, Montreal, QC Canada

**Keywords:** Obesity, Weight-loss, Microbiome, Metabolome, Gut barrier

## Abstract

**Background:**

Obesity remains a global health challenge, and responses to lifestyle-based weight-loss interventions are heterogeneous. Here, we evaluate a one-year structured lifestyle program in 50 adults with obesity (mean BMI 42 ± 7.0 kg/m^2^), integrating clinical, microbiome, and metabolomic profiling, to identify predictors of weight-loss success and metabolic improvement (ClinicalTrials.gov: NCT01344525). The intervention included a 3-month very low-calorie formula diet (approximately 850 kcal/day), a 3-month transition phase from a formula diet to a balanced diet (approximately 1000 kcal/day), and a 6-month maintenance period in which the participants followed a balanced diet (gradually increasing to a maximum of 2000 kcal/day).

**Results:**

Following the intervention, the participants exhibited marked reductions in body weight, body fat percentage, C-reactive protein, and glycated hemoglobin. Longitudinal analyses revealed that shifts in the gut microbiota composition were associated with changes in clinical and anthropometric data, as well as gut barrier function. An increased abundance of *Lachnospiraceae* was associated with improved gut barrier function; the relationship was mediated by fecal butyrate and propionate. Multivariate analyses revealed that serum baseline levels of diacylphosphatidylcholine C40:1 predicted postintervention BMI, indicating that this metabolite could serve as a biomarker of weight loss success. A random forest model incorporating baseline microbial and clinical features was used to predict weight loss and clinical improvements with high accuracy.

**Conclusions:**

Our findings elucidate the interplay between the gut microbiota and host metabolism during weight loss and highlight the potential utility of baseline profiling to achieve a high success rate in personalized obesity treatment.

Video Abstract

**Supplementary Information:**

The online version contains supplementary material available at https://doi.org/10.1186/s40168-026-02501-x.

## Introduction

The prevalence of obesity and its associated comorbidities has reached epidemic proportions almost worldwide, necessitating improved strategies for prevention and treatment [1]﻿. Self-motivated weight-loss efforts rely on calorie restriction and exercise but often fail because of the high level of discipline needed or inadequate approaches. As an alternative, guided interventions include bariatric surgery or guided weight-loss programs, which usually comprise a low-calorie diet, behavioral training, or pharmacotherapy. While bariatric surgery is effective, it carries risks such as sarcopenia due to rapid weight loss; vitamin deficiency; and digestive complications, such as reflux and rapid gastric emptying (commonly known as dumping syndrome) [2]. Multimodal programs, including formula diets and increased physical activity, offer an effective alternative to bariatric surgery, inducing rapid initial weight loss with minimal side effects [3, 4]. However, compared with surgical interventions, such weight-loss programs require greater self-discipline, time, and financial resources to achieve sustained weight loss [5]. Individuals who fail to achieve results on their first weight-loss attempt are frequently discouraged from engaging in future efforts [6]. One way to increase the efficiency of such programs would be to identify in advance those individuals who would benefit from such an intervention.

Weight-loss success varies between individuals following the same intervention and depends on a complex interplay among environmental, genetic, neural, and endocrine factors [7], as well as the gut microbiota [8]. In detail, interindividual weight reduction variability likely depends on the baseline gut microbiota composition, as demonstrated in previous work by us [9] and others [10]. The mechanisms by which the gut microbiota affects obesity development and treatment remain incompletely understood and are often based on preclinical data [7].

Obesity is associated with altered gut microbiota composition and impaired gut barrier function [11, 12], resulting in increased translocation of endotoxins from the intestine to the liver, triggering inflammatory processes in the liver and beyond [13]. These processes can result in low-grade inflammation that fosters the development of noncommunicable diseases such as type 2 diabetes, atherosclerosis, and cancer [14, 15]. While overeating and obesity as well as fiber-deprived diets promote gut barrier dysfunction and low-grade inflammation, caloric restriction and a high-fiber diet can reverse these effects [16, 17]. Apart from microbiota changes and low-grade inflammation, the serum metabolome has also been proposed as a potential predictor of clinical outcomes, including weight-loss success [18–21].

We previously reported improved gut barrier function, measured using the lactulose-mannitol test and fecal zonulin, in 27 participants who completed a one-year weight-loss program [22]. We also previously showed, in small sample sizes (*n* = 15 and 16), that weight loss through this structured one-year program was associated with an increased abundance of *Akkermansia* and that the baseline microbiota may predict the success of the intervention [9, 23].

**Fig. 1:**
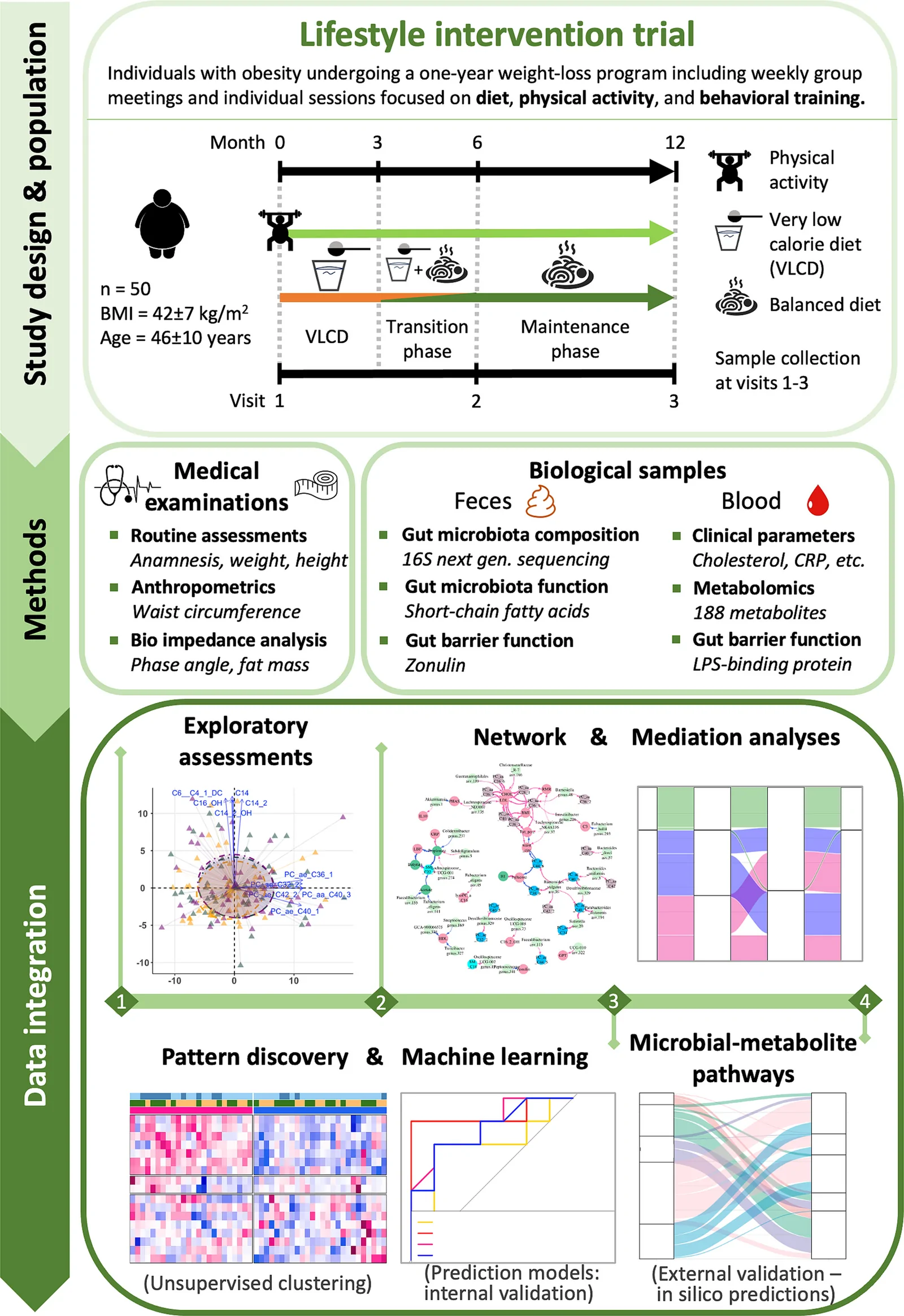
Study design and analysis overview. Fifty adults with obesity underwent a one-year weight-loss program. Clinical assessments, along with blood and fecal sampling, were conducted at baseline (month 0), month 6, and month 12. Multi-omic analyses were performed to identify potential predictors of successful weight loss and investigate the underlying mechanisms involved. CRP, C-reactive protein; LPS, lipopolysaccharide

Here, we expand the scope of the analysis by examining not only baseline but also longitudinal data on a comprehensive set of clinical, gut microbiota, and serum metabolome parameters in a cohort of 50 individuals with obesity undergoing a standardized one-year lifestyle intervention program (Fig. 1). In addition to gathering new insights into the mechanisms by which the intervention affects weight loss, our goal is to evaluate whether baseline multi-omic profiles can predict weight loss and clinical improvements, laying the groundwork for personalized nutrition.

## Results

### Beneficial health effects of the weight-loss intervention

**Fig. 2:**
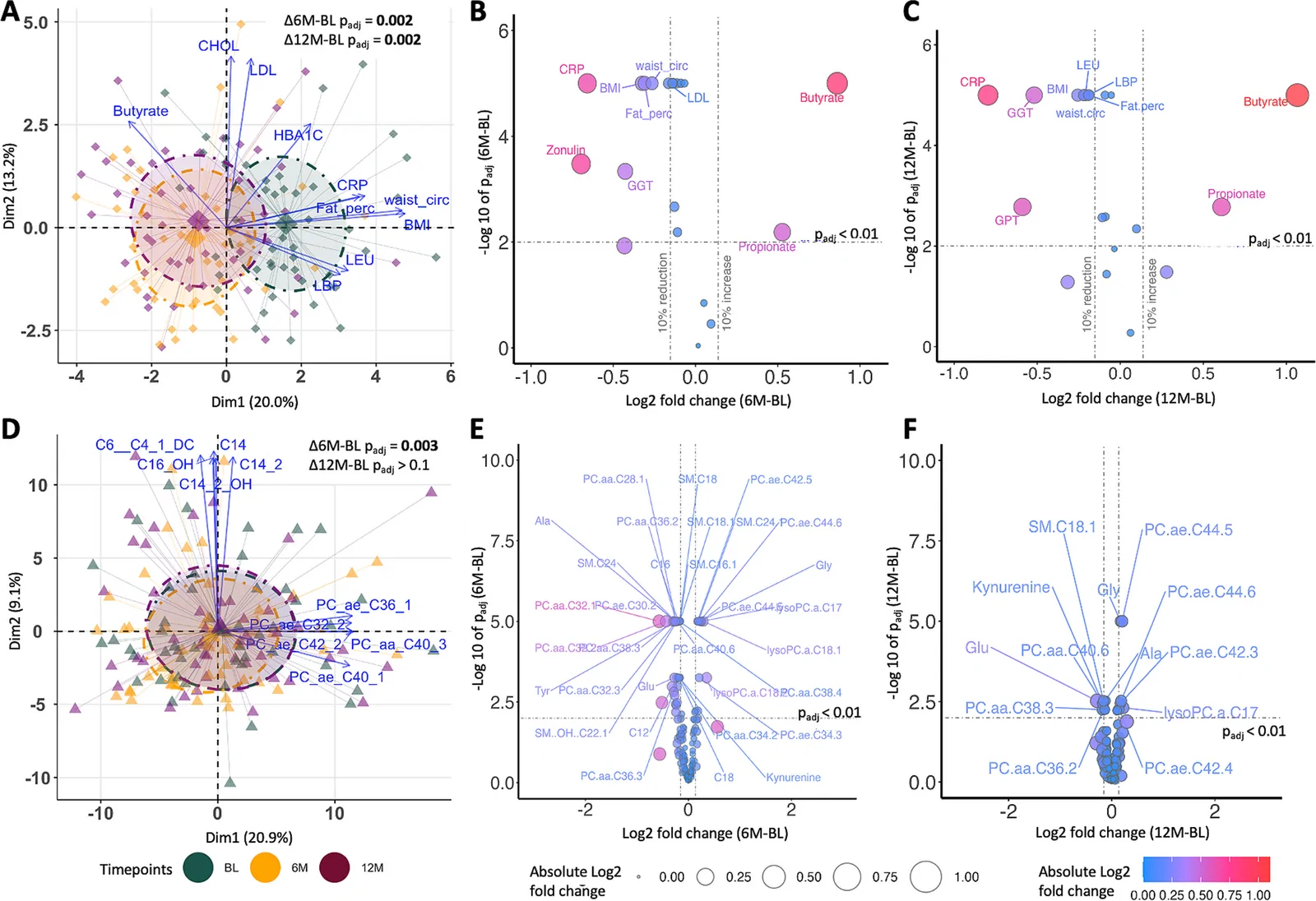
Effects of the lifestyle intervention on clinical outcomes (A-C) and serum metabolomics (D-F). **A**, **D,** Principal component analysis (PCA) biplots illustrating the distribution of individuals (points) and the contribution of variables (arrows) for the clinical (panel A, *n* = 141) and metabolomic (panel D, *n* = 150) data. The top 10 contributing variables are displayed as blue arrows. Ellipses represent the group covariance structure. The PCA was performed with Euclidean distance, and the data were scaled before transformation. An ordination analysis was performed using a permutational multivariate analysis of variance (PERMANOVA), adjusted for sex and individual variations. **B-C and E–F**, Volcano plots showing the parameters that changed between baseline (BL) and months 6 (6 M) and 12 (12 M) (linear mixed models adjusted for age and sex). These panels visualize the data shown in detail in the Supplementary Excel File

Using clinical data from 50 individuals with obesity (mean age of 46 ± 9.6 years and mean BMI of 42 ± 7.0 kg/m^2^; Supplementary Excel File), we performed a multivariable analysis to investigate the possible effects of the weight loss program. There was a distinct difference in the clinical multivariable dataset between baseline (BL) and 6 months (6 M), as well as between BL and 12 months (12 M), as assessed by permutational multivariate analysis of variance (PERMANOVA; Fig. 2A).

Specifically, significant decreases in BMI, body fat mass percentage, and waist circumference were observed after 6 and 12 months (all p_adj_ < 0.001, linear mixed models; Fig. 2B,C). Additionally, the plasma levels of total cholesterol, low-density lipoprotein (LDL) cholesterol, and C-reactive protein (CRP) decreased after 6 and 12 months (all p_adj_ < 0.001). In addition, the plasma level of the gut barrier biomarker lipopolysaccharide binding protein (LBP) decreased after 6 and 12 months (p_adj_ < 0.001), and the fecal level of zonulin decreased after 6 months of intervention (p_adj_ < 0.001) and after 12 months (p_adj_ = 0.052). Moreover, increases in the fecal levels of the short-chain fatty acids (SCFAs) butyrate and propionate were observed after 6 and 12 months (p_adj_ < 0.010), and the content of the SCFA acetate increased after 12 months of intervention (p_adj_ = 0.033; Fig. 2B,C).

### The weight-loss intervention changed the serum metabolome

A targeted serum metabolomics assessment revealed a significant overall difference after 6 months but not after 12 months of intervention (PERMANOVA; Fig. 2D). In particular, we observed increases in the serum levels of the amino acids asparagine, glycine, and serine as well as several lysophosphatidylcholines (all p_adj_ < 0.05, linear mixed models) and decreases in alanine and glutamate, as well as alpha-aminoadipic acid, kynurenine, phosphatidylcholines, and sphingomyelins, after 6 months (all p_adj_ < 0.05, Fig. 2E,F).

### The weight-loss intervention changed the gut microbiota composition

**Fig. 3:**
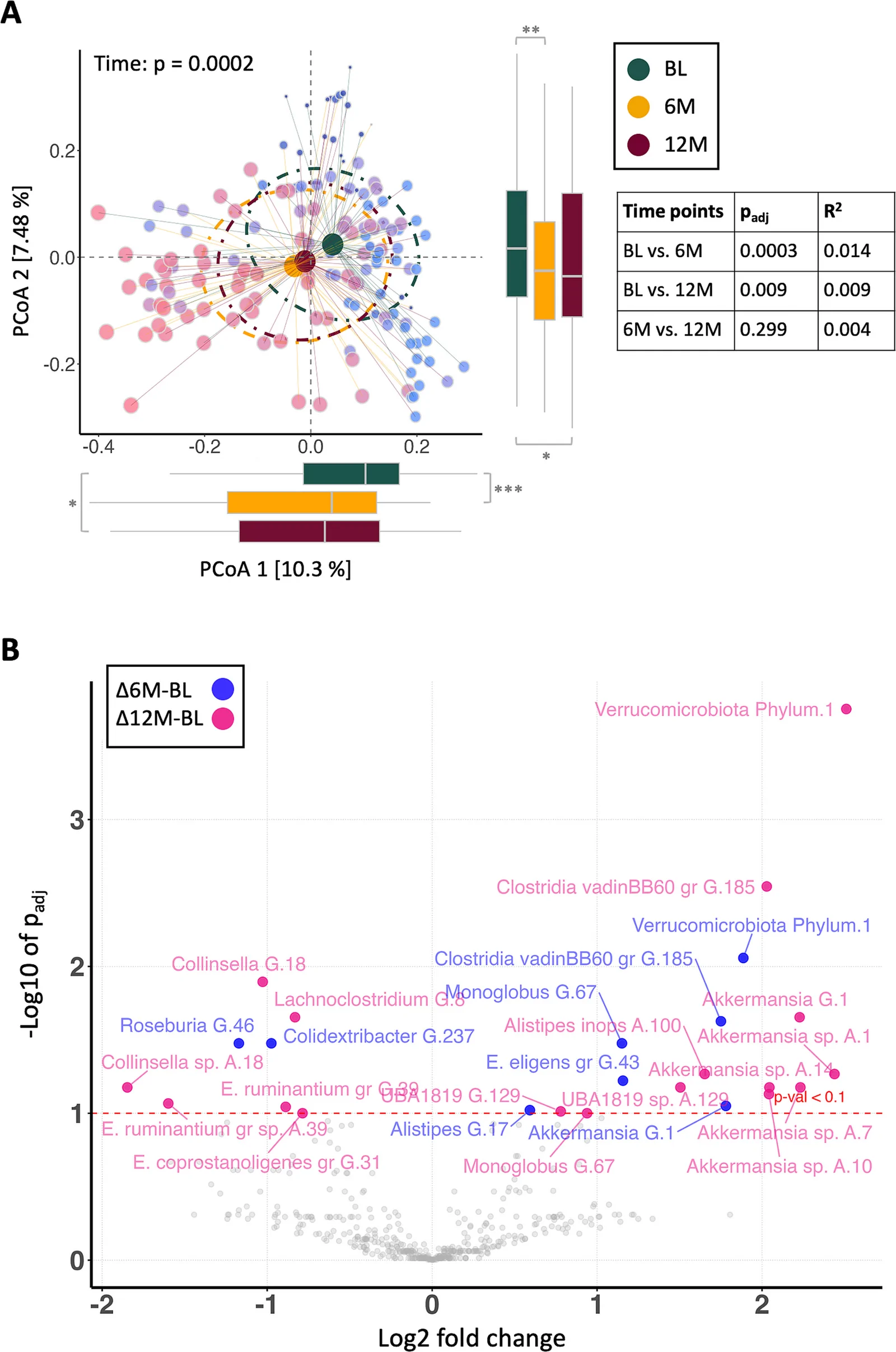
Gut microbiome changes. **A** Principal coordinate analysis (PCoA) plot depicting the beta diversity of the gut microbiome on the basis of Bray‒Curtis dissimilarity at baseline (BL), month 6 (6 M), and month 12 (12 M) (*n* = 141). An ordination analysis was performed using a permutational multivariate analysis of variance (PERMANOVA), adjusted for sex and individual variations. Boxplots on the bottom and on the right show PCoA1 and PCoA2 in detail (linear mixed models; *p < 0.05, **p < 0.01, ***p < 0.001). **B** Volcano plot showing gut microbiota parameters that changed between BL, 6 M, and 12 M (linear mixed models)

Using 16S rRNA sequencing, we found no effect of the intervention on gut microbial alpha diversity (all p_adj_ > 0.1, linear mixed models; Figure S1A). PERMANOVA on Bray‒Curtis dissimilarity revealed that there were significant differences in beta diversity among the time points (overall time effect: *p* = 0.0002) (Fig. 3A). Accordingly, when we separately examined PCoA1 and PCoA2, we detected differences between BL and month 6 as well as between BL and month 12 (linear mixed models; Fig. 3A). In line, PCoA based on weighted and generalized UniFrac distances showed significant differences among the time points (overall time effects: *p* = 0.002 and *p* = 0.0002, respectively) (Figure S1B,C). Accordingly, when we separately examined PCoA1 and PCoA2, we detected differences between BL and month 6 as well as between BL and month 12 (linear mixed models; Figure S1D,E).

We observed changes in the ratios of bacterial taxa, such as an increase in the *Akkermansia/Prevotella* ratio in the first 6 months (p_adj_ = 0.002, linear mixed models), and this increase over BL persisted for at least 12 months (p_adj_ = 0.02) (Figure S2A). There was no difference in the *Firmicutes/Bacteroidota* ratio among the time points (p_adj_ > 0.1; data not shown). In a more detailed examination of bacterial taxa, we detected a greater relative abundance of the phylum Verrucomicrobiota at month 6 (p_adj_ < 0.001) and month 12 (p_adj_ = 0.009) than at BL (Fig. 3B, Figure S2B). Additionally, the relative abundances of the genera *Akkermansia, Monoglobus,* and *Clostridia vadin* and amplicon sequencing variants (ASVs) related to *Akkermansia* were greater at month 6 and month 12 than at BL (all p_adj_ < 0.1; Fig. 3B and Figure S2C).

### Clinical effects were closely associated with changes in the gut microbiome and serum metabolome

**Fig. 4:**
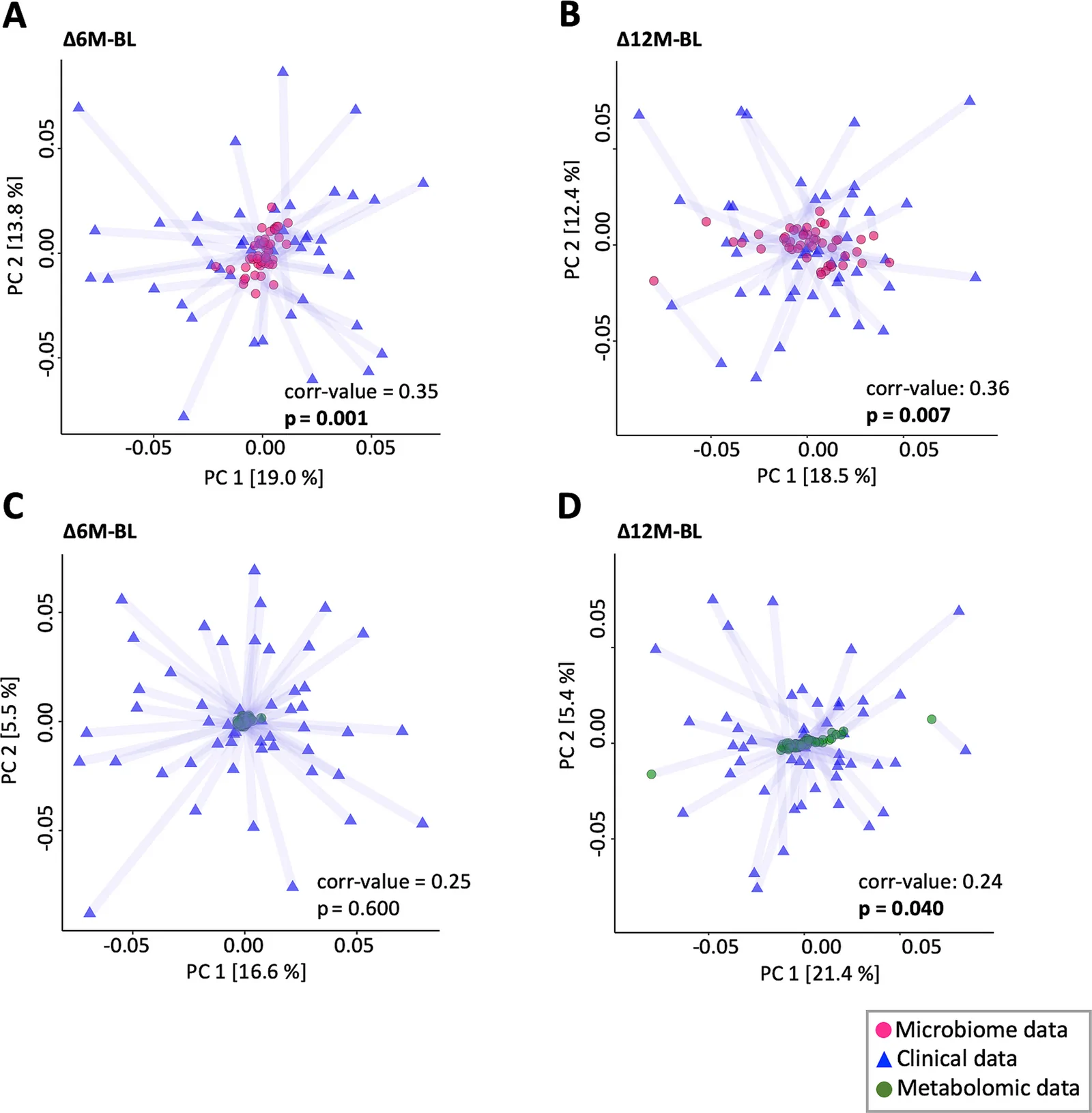
Anthropometric and clinical changes are strongly associated with changes in the gut microbiome and with the serum metabolome.** A**-**D** Procrustes analysis visualizing the alignment between the shifts in the microbiome, clinical, and metabolomic datasets (*n* = 47). Here, shorter lines indicate greater concordance

We hypothesized that changes in clinical parameters were linked to changes in the gut microbiome and the serum metabolome, and we tested this hypothesis using Procrustes analyses. For these analyses, we calculated the shift (BL values subtracted from the values at month 6 or month 12, shown as 6 M-BL and 12 M-BL). Our findings revealed a significant association between changes in the gut microbiota composition and changes in the clinical parameters for both 6 M-BL (*p* = 0.001, Procrustes analysis; Fig. 4A) and 12 M-BL (*p* = 0.007; Fig. 4B).

We further examined whether the shift in the serum metabolome between the BL and 6 M/12 M groups was associated with shifts in the clinical parameters. Here, no significant association was observed for 6 M-BL (*p* = 0.6; Fig. 4C). Interestingly, a significant association was found for 12 M-BL (*p* = 0.040; Fig. 4D). To explore these observed associations in more detail, we performed subsequent network and mediation analyses.

### Network and mediation analyses decipher how bacterial and metabolic features are associated with clinical outcomes

**Fig. 5:**
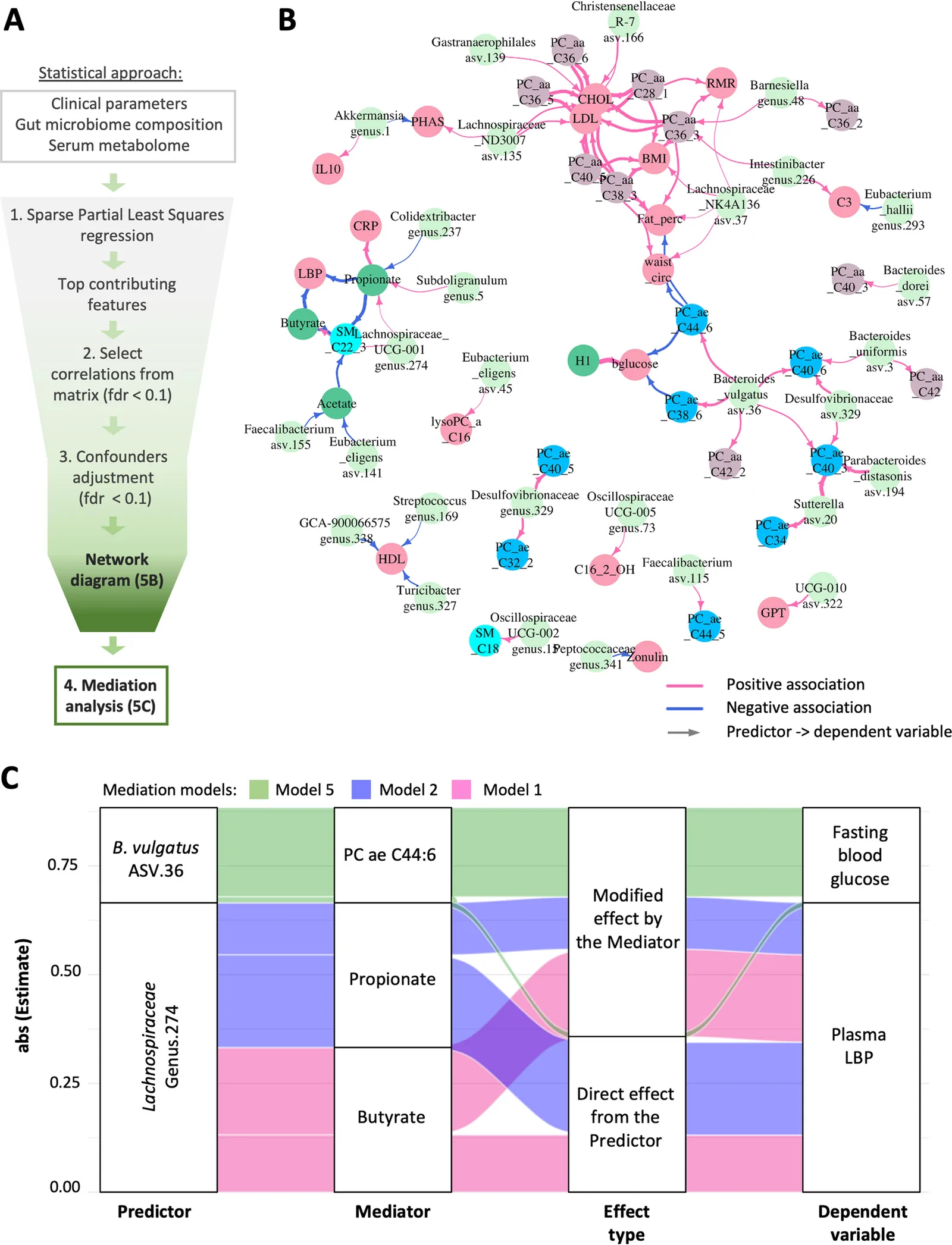
Integration of the multi-omic datasets reveals candidate features linking changes in the microbiome to changes in clinical outcomes.** A** Flow diagram of the statistical approach leading to the results shown in panels B and C, as described in detail in the Supplementary Methods.** B** Network visualization of the significant associations among microbial taxa (CLR-transformed ASVs), metabolomics, and clinical outcomes for the shift between baseline and month 6 (*n* = 47–50). Microbiome features act exclusively as predictors, whereas clinical features act solely as outcomes. Metabolites serve as either predictors or outcomes depending on their role in the regression model. The colors of the circles indicate the different datasets. **C** Results of the mediation analysis. Mediation models were constructed in the following direction: microbiome (predictor) → metabolite (mediator) → clinical variable (outcome). Only models with significant indirect and total effects (p_adj_ < 0.1, 500 bootstrap replications) are shown (*n* = 47–50). This figure summarizes the main findings, which are shown in detail in the Supplementary Excel File

We employed sparse partial least squares regression to identify single key clinical, metabolomic, and microbiome features contributing to the associations found in the Procrustes analyses. Based on the biological assumption that lifestyle changes induce alterations in the gut microbiota and serum metabolome, which in turn affect clinical parameters, we treated the microbiome and metabolome as predictors and the clinical parameters as outcomes. Under stringent selection criteria (Fig. 5A and Supplementary Methods), the final set of relationships was visualized as networks (6 M-BL, Fig. 5B; 12 M-BL, Figure S3). In the 6 M-BL network (Fig. 5B), we found three main clusters of relationships, where the nodes were connected by arrows with consistent directionality, suggesting distinct functional groupings. These clusters were characterized by the following features: 1) BMI, body fat percentage, resting metabolic rate, cholesterol, LDL cholesterol, and serum diacylphosphatidylcholines; 2) *Bacteroides vulgatus* (ASV.36), fasting blood glucose levels, and serum alkylacylphosphatidylcholines; and 3) fecal butyrate and propionate levels and plasma LBP and CRP levels. Although these features exemplify the main clusters, numerous other interconnections were found (Fig. 5B).

To further explore the relationships of interest within these clusters, we manually selected a set of main candidates for subsequent mediation analyses, hypothesizing that selected metabolites mediate the effects of the gut microbiota on clinical outcomes. As a result, 10 mediation models were generated, of which only three models showed both significant mediated and total effects and were therefore considered meaningful (Fig. 5C and Supplementary Excel File).

As shown in Fig. 5C, models 1 and 2 examined the role of butyrate and propionate as mediators between *Lachnospiraceae* (genus 274) and plasma LBP. In both models, the indirect effects (ACMEs) were significant (both p_adj_ = 0.060). Notably, the direct effects (ADEs) were not significant in either model (p_adj_ ≥ 0.1), suggesting that *Lachnospiraceae* (genus 274) influences LBP primarily through butyrate and propionate. The total effects in both models remained significant in the model with all variables included (both p_adj_ = 0.060), supporting the role of the two SCFAs in linking *Lachnospiraceae* (genus 274) to LBP.

In the third model found to be relevant, acylalkylphosphatidylcholine (PC ae) C44:6 was tested as a mediator between *Bacteroides vulgatus* (ASV.36) and fasting blood glucose levels. The indirect effect (ACME) was significant (p_adj_ = 0.060), indicating that PC ae C44:6 mediates this relationship. The direct effect (ADE) was not significant (p_adj_ = 0.911), indicating that the effect of *Bacteroides vulgatus* (ASV.36) on blood glucose was predominantly indirect. The total effect remained significant (p_adj_ = 0.060), emphasizing the potential role of PC ae C44:6 as a mediator between *Bacteroides vulgatus* (ASV.36) and blood glucose levels.

The results of the mediation analysis highlight potential mechanistic links among the gut microbiome, metabolic intermediates, and clinical outcomes, warranting further exploration.

### Baseline features predict the effect of the intervention on single clinical outcomes

**Fig. 6:**
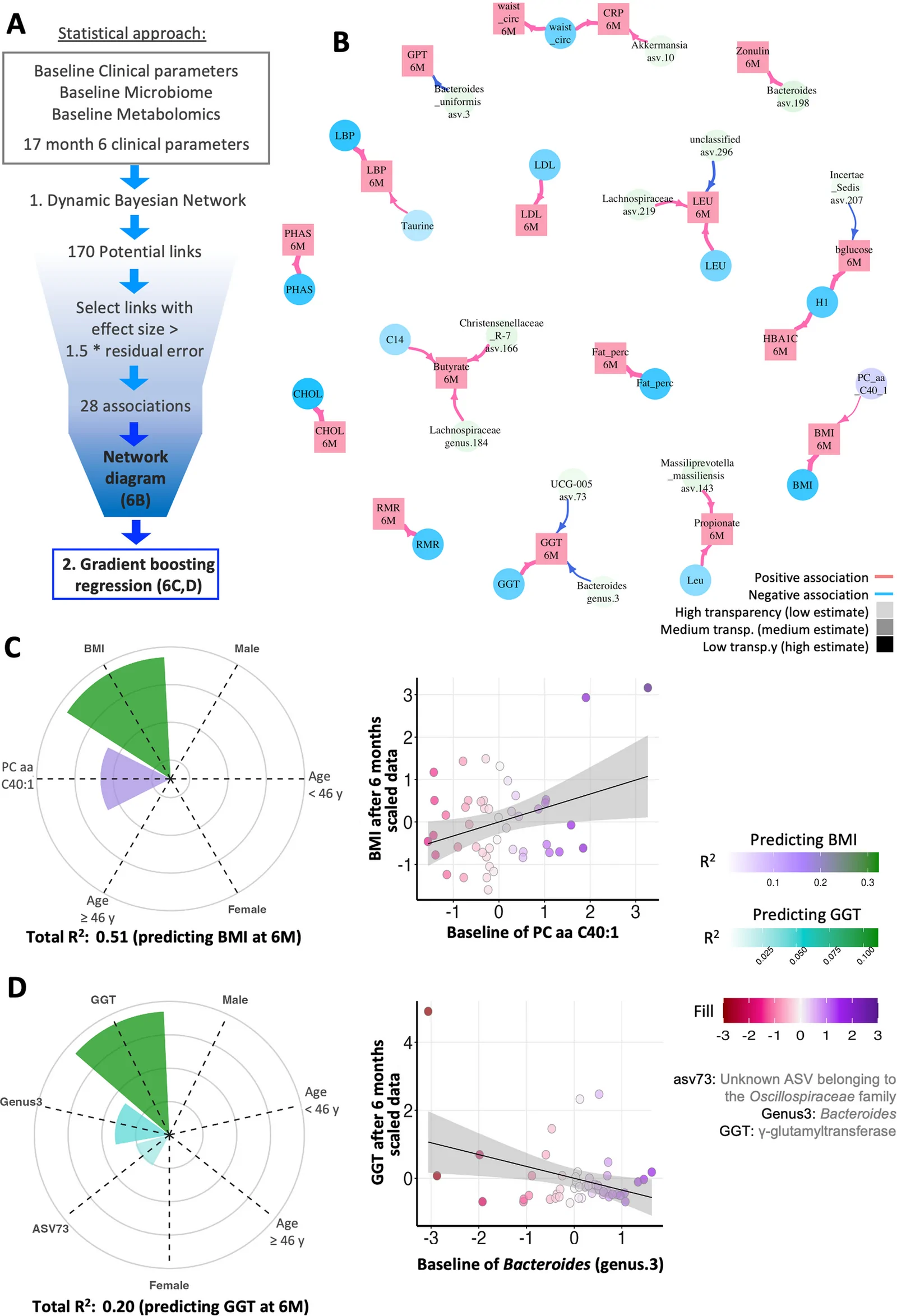
Baseline microbiome and metabolome features predict the effect of the intervention on single clinical outcomes. **A** Flow diagram of the statistical approach leading to the results shown in panels B to D, as described in detail in the Supplementary Methods.** B** Dynamic Bayesian network showing the baseline microbiome and metabolome features that predict the clinical parameters after 6 months (*n* = 47). Circles denote baseline predictors, and squares denote significantly influenced postintervention outcomes. The network, filtered using a residual threshold of 1.5, was reduced to 28 connections for visualization. **C D** Circular bar plots (left side) showing the results of the gradient boosting regression, presenting the coefficient of determination (R^2^) for each baseline predictor influencing a selected clinical parameter at month 6. Building on the variables identified in panel B, the model was trained on 70% of the samples where the values of the features were determined by the other baseline features rather than solely their own. The bar height indicates the predictor’s explanatory power (R^2^), and the color gradient reflects the range of effect magnitudes (from low to high R^2^). The dashed radial lines form lollipop shafts representing each predictor’s modeled influence. Scatter plots (right side) showing the linear relationship between baseline features and 6-month outcomes for the predictors selected in panel B. Each dot represents an individual observation

We tested whether particular baseline features, such as the baseline microbiome or metabolome data, could predict changes in clinical outcomes after 6 months of intervention by a multistep statistical approach (Fig. 6A and Supplementary Methods). Specifically, 641 baseline features derived from the clinical, microbiome and metabolomic datasets served as predictors. A set of 17 clinical features was analyzed as outcomes, comprising those features that showed significant changes after 6 months of intervention (Supplementary Excel File). We modeled possible associations between predictors and outcomes to identify predictive relationships.

Using the Bayesian information criterion for model selection, the dynamic Bayesian network identified 170 potential relationships between the baseline predictors and the outcome features after 6 months. To refine the results and ensure robustness, we selected only relationships with an estimated effect size at least 1.5 times greater than the residual error. This filtering process reduced the final network to 28 associations, which were visualized as a structured network (Fig. 6B). These results highlight key baseline determinants that may have driven the observed clinical changes, providing insights into predictive factors shaping long-term health outcomes. For example, we found that the baseline abundance of *Bacteroides uniformis* (ASV.3) was strongly associated with plasma γ-glutamyltransferase levels after 6 months and that the baseline serum levels of diacylphosphatidylcholine (PC aa) C40:1 were strongly associated with BMI after 6 months.

To further validate these key relationships identified in the dynamic Bayesian network, we applied gradient boosting regression. Here, the models were selected from the network on the basis of their structure, ensuring that the dependent variable was influenced by baseline features other than its own. After these models were adjusted for sex and age, only two models were significant, with high R^2^ values (> 0.15) and low mean absolute errors (< 0.5). In the first model, baseline PC aa C40:1 was a strong predictor of BMI after 6 months (total R^2^ = 0.511) (Fig. 6C). In the second model, the baseline abundance of *Bacteroides uniformis* (ASV.3) was a strong predictor of the liver function marker γ-glutamyltransferase after 6 months (total R^2^ = 0.198), demonstrating a negative association (Fig. 6D). These findings indicate that the baseline microbiome and metabolomic markers can predict clinical outcomes after intervention, a finding that we further investigated.

### The effect of the intervention on clinical outcomes is individualized

**Fig. 7:**
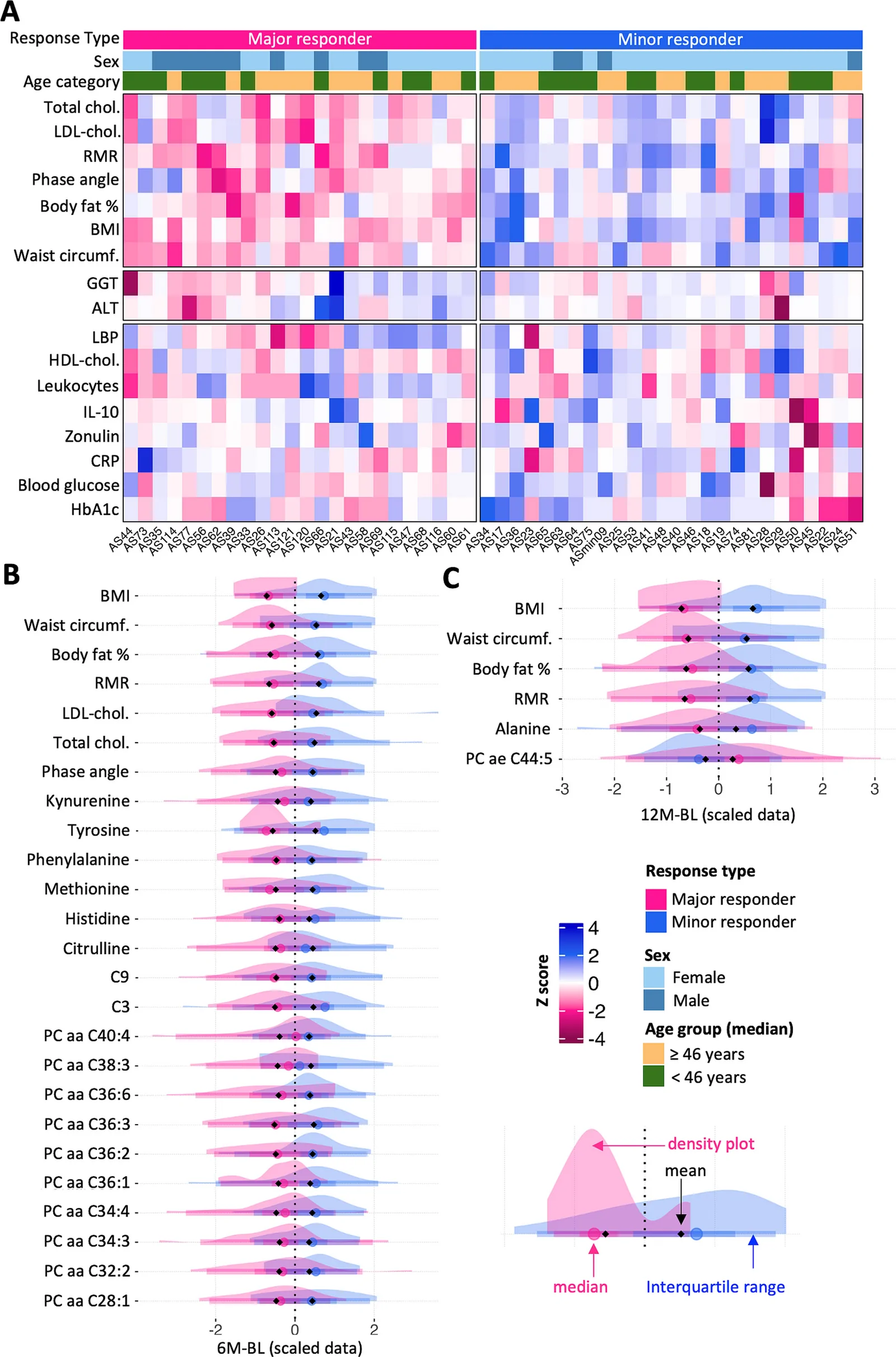
The effect of the intervention on clinical outcomes is individualized. **A** Heatmap visualizing the standardized shifts in clinical features (excluding SCFA data) between baseline (BL) and month 6 (6 M; shown as 6 M-BL) (*n* = 50). The data were scaled and centered prior to clustering. Unsupervised hierarchical clustering was performed using Ward’s method on Euclidean distances, resulting in two response groups: major responders (pink) and minor responders (blue). The rows were divided into three clusters on the basis of the results from the hierarchical clustering. **B**, **C** Density plots showing the features that differentiate major and minor responders. Only features with a p_adj_ < 0.05 are shown (*n* = 50)

Although the mean values improved for almost all clinical features during the study (Supplementary Excel File), we found substantial individual variability (Fig. 7A). To explore this heterogeneity, we aimed to identify distinct response patterns among participants. We applied an unsupervised clustering approach based on the shifts in the clinical features (not including SCFAs) for 6M-BL. Here, all clinical features were weighted equally because our aim was to evaluate overall improvements in clinical outcomes, rather than solely focusing on a single feature, such as body weight. This analysis revealed two distinct clusters: 24 major and 26 minor responders (Fig. 7A).

To further understand the individual responses, we applied linear models adjusted for sex and age to compare the shifts in the clinical and metabolomic markers between major and minor responders for both 6M-BL (Fig. 7B) and 12M-BL (Fig. 7C). For 6M-BL, several clinical and metabolic features significantly distinguished the two groups. Major responders exhibited greater improvements than minor responders in BMI, total cholesterol, LDL, fat percentage, phase angle, resting metabolic rate, and waist circumference (all p_adj_ < 0.003). In addition to these classical clinical features, several unexpected features, such as selected amino acids, acylcarnitines and, in particular, phosphatidylcholines, were found to distinguish the groups (Fig. 7B). For 12M-BL, fewer features remained significantly different, with BMI, fat percentage, resting metabolic rate, and waist circumference continuing to distinguish the groups (all p_adj_ < 0.001) (Fig. 7C).

Importantly, there was no difference in compliance, measured by the weekly meeting attendance rate, between major and minor responders after 6 months (*p* = 0.578, Mann–Whitney test) or 12 months (*p* = 0.512). All participants were closely monitored regarding diet, exercise, and other lifestyle factors during the weekly meetings. Therefore, the different clinical outcomes observed in major and minor responders are unlikely to be explained by behavioral or lifestyle differences between individuals. Furthermore, there was no baseline difference in gut microbiome composition between major and minor responders (all p_adj_ > 0.1; Figure S4).

### Baseline features predict individual responses to the lifestyle intervention

To identify baseline features capable of predicting whether an individual would be a major or minor responder, we used a targeted feature selection strategy based on the results of the prior network-based analyses, resulting in 31 baseline features as predictors, including selected clinical, metabolomic, and microbiome variables. We evaluated separate models for each main dataset (clinical parameters, gut microbiota composition, and serum metabolomics). Moreover, we used data subsets such as selected serum metabolites shown to have high predictive power in the previous network analysis, as well as combined models of these datasets (Supplementary Excel File). We trained multiple random forest classification models on 70% of the total dataset of 50 individuals and tested the model’s performance on the remaining 30%.

**Fig. 8:**
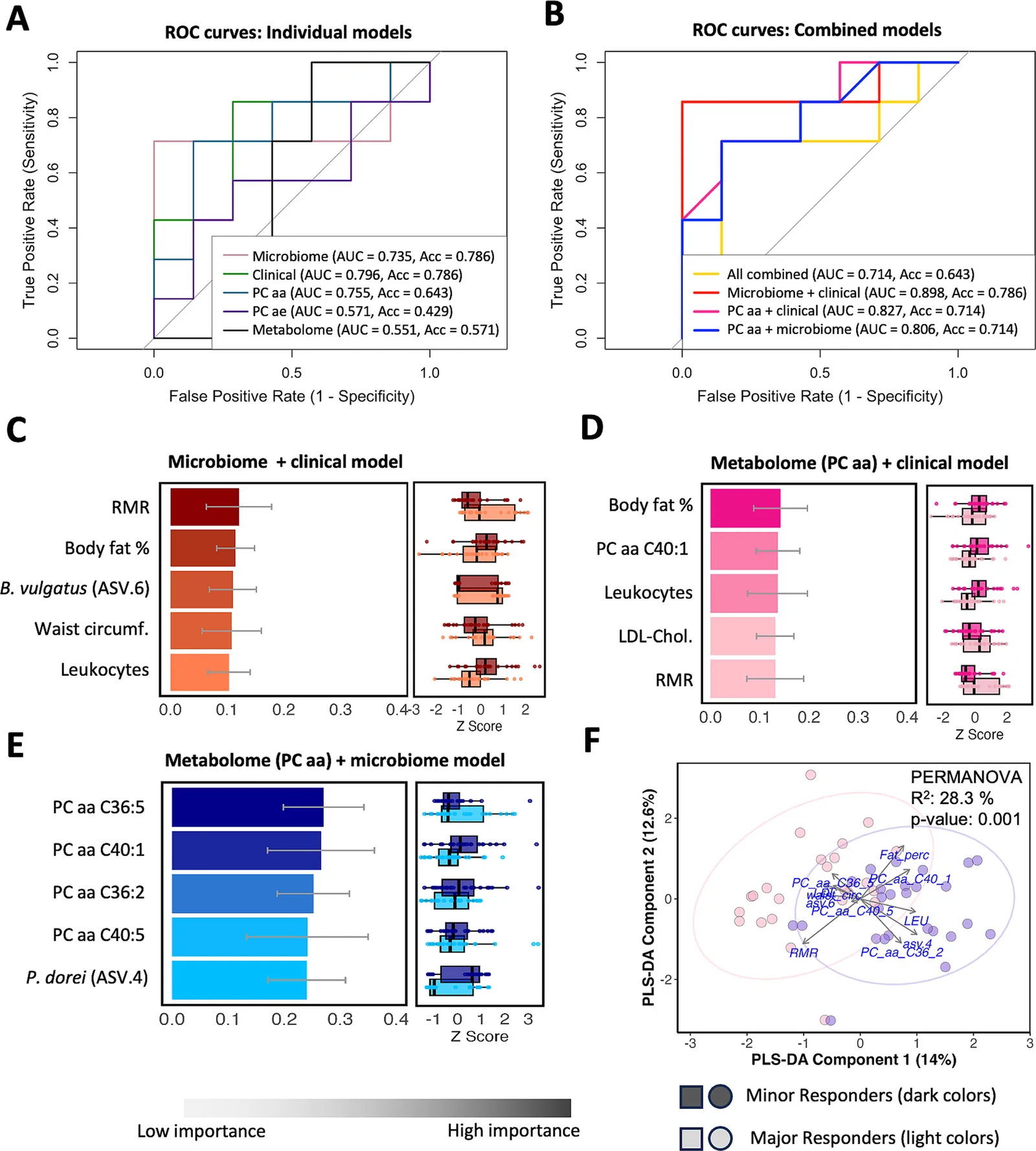
Baseline characteristics predict individual responses to the lifestyle intervention.** A**, **B** Receiver operating characteristic (ROC) curves of random forest classification models predicting responder types (major vs. minor responders) using different baseline feature sets (*n* = 50). The ROC curves show the model performance in a tenfold cross-validation framework, where the area under the curve (AUC) and accuracy (%) are reported for each model. PC aa, diacylphosphatidylcholine; PC ae, acylalkylphosphatidylcholine. **C-E** Bar plots showing the top five most important predictive features, ranked by the mean decrease in the Gini coefficient. The error bars represent the standard deviation of feature importance scores across cross-validation folds. On the right side of each bar plot is a horizontal boxplot that shows the distribution of Z-scored values for the top five important features in each combined model, comparing major and minor responders (*n* = 50). **F** Partial least squares discriminant analysis visualizing sample separation on the basis of the top selected features from the combined models (*n* = 50). The ellipses represent 95% confidence intervals around major and minor responders. The arrows indicate the contribution of each variable to class separation

Individually, the microbiome, metabolome, and clinical models showed moderate predictive performance, with AUC values ranging from 0.551 (metabolomics) to 0.796 (clinical parameters) (Fig. 8A). On the basis of these findings, we further tested combined models incorporating different combinations of the top-performing feature groups to assess whether the predictive performance could be improved. Among the combined models, the microbiome and clinical model achieved the best performance (AUC 0.898), followed by the serum metabolome and clinical model which had an AUC of 0.827 (Fig. 8B). Figure 8C-E shows the top five features for each model (left side), as well as boxplots showing the distribution of these features between major and minor responders (right side). Notably, clinical features such as resting metabolic rate, body fat percentage and plasma leukocytes appeared repeatedly across the different models, as did PC aa C40:1, consistently highlighting their predictive value.

To visualize how well the selected features separated major and minor responders, we applied partial least squares discriminant analysis (Fig. 8F). This multivariate technique reduces the dimensionality of the data while maximizing group separation. Components 1 and 2 explained 14% and 13% of the variance in the predictor space, respectively. A PERMANOVA based on the two-component partial least squares discriminant analysis space confirmed a significant separation between major and minor responders (R^2^ = 28.3%, *p* = 0.001), supporting the presence of distinct baseline profiles between the responder types (Fig. 8F). Taken together, these results highlight the strong predictive power of a multi-omic approach to predict individual weight-loss responses from baseline profiles.

### Unraveling potential microbial interactions to investigate metabolic mechanisms

**Fig. 9:**
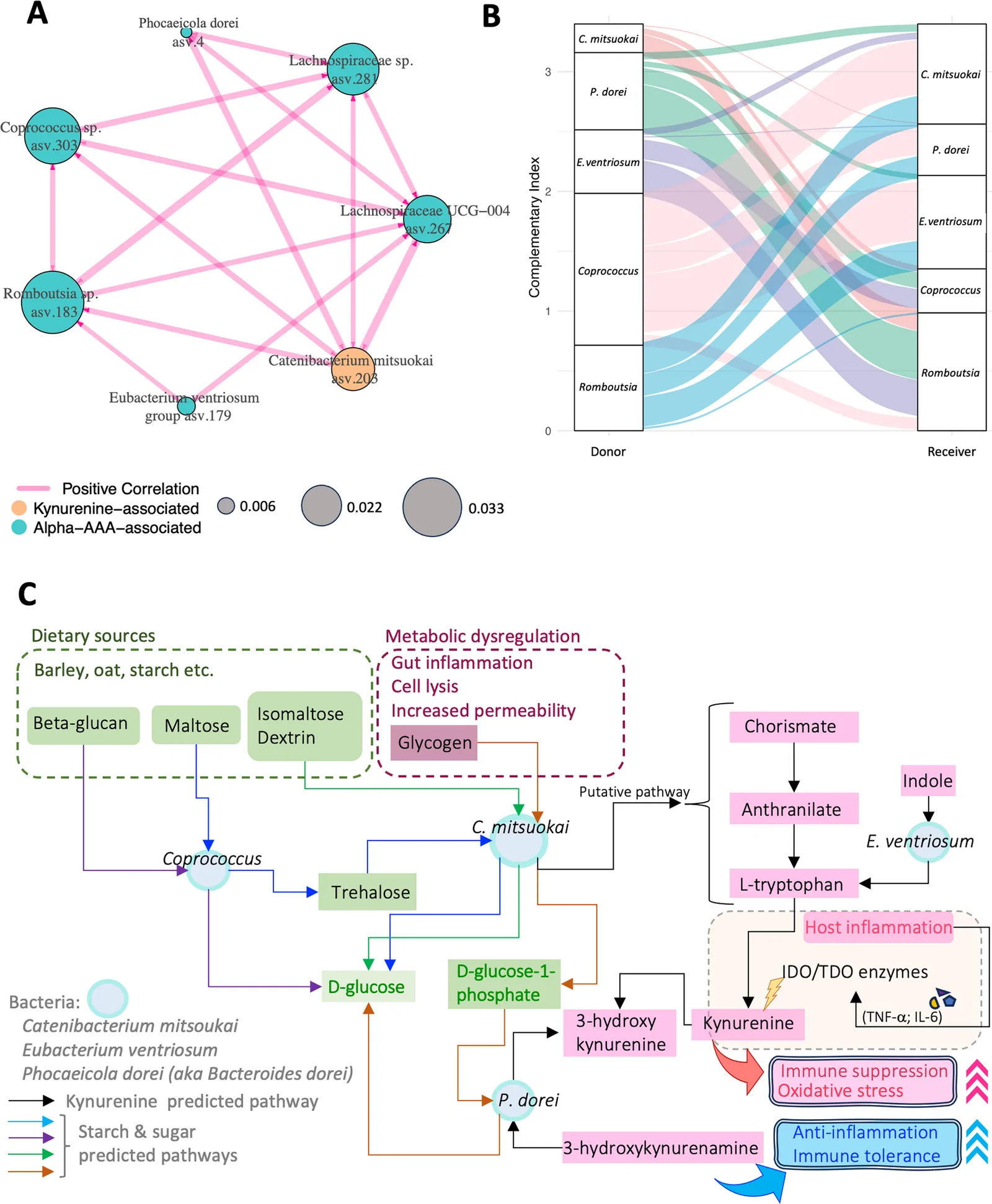
Investigating potential microbial interactions to better understand the underlying metabolic mechanisms.** A** Microbiome association network of seven bacteria identified by elastic net regression. The node size represents the size of the regression coefficient. The orange nodes indicate kynurenine-associated taxa, the blue nodes represent alpha-aminoadipic acid** (**alpha-AAA)-associated taxa, and the pink edges reflect significant positive correlations between microbial shifts from baseline to month 6 (shown as 6 M-BL). **B** Flowchart of microbial metabolic interactions based on MicrobiomeNet and KEGG-based genome-scale modeling. This diagram shows the predicted microbial interactions involving tryptophan metabolism and carbohydrate degradation/cross-feeding among the taxa identified in the regression analysis. The width of each band corresponds to the level of metabolic support provided by one species to another. **C** Putative schematic overview of microbial interactions linking kynurenine metabolism and carbohydrate cross-feeding. Arrows represent predicted metabolic interactions inferred from genome-scale modeling. IDO, indoleamine 2,3-dioxygenase; TDO, tryptophan 2,3-dioxygenase

Among the biogenic amines measured in this study, alpha-aminoadipic acid (alpha-AAA), kynurenine, and putrescine were the only metabolites that changed significantly (*p*_*adj*_ < 0.05) after the 6-month weight-loss intervention (Supplementary Excel File). Alpha-AAA and kynurenine are host-derived metabolites strongly associated with obesity, oxidative stress, and immune dysregulation [24, 25], whereas putrescine is linked to gut microbial polyamine metabolism [26]. To explore whether shifts in these metabolites were associated with specific microbial taxa, we applied elastic-net regression to centered log-ratio (CLR)-transformed ASV shift data. Although putrescine was included in this screening, no ASV predictor was retained for putrescine under the selected model which is why we did not focus on this metabolite in the subsequent analyses. In contrast, alpha-AAA and kynurenine retained ASV predictors, resulting in seven ASVs associated with changes in either metabolite. An abundance-based correlation analysis revealed solely positive associations among these taxa (Fig. 9A).

Since the abundance-based correlation analysis alone could not determine the nature of the ecological interactions and functional relationships between these bacteria, we computed pairwise metabolic complementarity scores using MicrobiomeNet, an open-access database used to link microbial genomes to bacterial metabolite production. These scores were visualized in a directional flow diagram, where the width of each band represents the fraction of nonessential compounds that can be synthesized by one species and potentially utilized by another as a key metabolic input (Fig. 9B). These analyses are based on reconstructed metabolic networks and represent computational predictions that were not experimentally validated in this study. They are intended to generate novel hypotheses for future work. This external analysis indicated strong cooperative potential within the bacterial groups, with *Coprococcus* sp., *Romboutsia*, and *Phocaeicola dorei* (formerly *Bacteroides dorei*) emerging as potentially important contributors within the network. These findings suggest potential metabolically coordinated microbial interactions that may adapt collectively in response to lifestyle changes.

### Potential microbial roles in the kynurenine pathway and carbohydrate degradation

In the previous analysis, we found that serum kynurenine levels decreased following the intervention (Fig. 2E,F and Supplementary Excel File) and that *Phocaeicola dorei* was a predictor of responsiveness to the intervention (Fig. 8E). To investigate the potential relationship between these findings, we constructed a putative microbial metabolic flowchart linking diet, gut bacteria, kynurenine, and carbohydrate degradation pathways on the basis of genome-scale metabolic modeling and KEGG-based annotations via MicrobiomeNet (Fig. 9C). These results suggest that *Catenibacterium mitsuokai* may encode enzymes for upstream tryptophan biosynthesis (e.g., chorismate to anthranilate), although complete de novo synthesis has not been fully confirmed. *Eubacterium ventriosum* was predicted of being capable of synthesizing L-tryptophan via conversion from indole, and *Phocaeicola dorei* likely catabolizes 3-hydroxykynurenine into 3-hydroxyanthranilate plus alanine. A potential immunological relevance of these predicted products requires further validation. This layered functional specialization highlights a potential cooperative network of microbial contributions to the host’s tryptophan metabolism.

In parallel, carbohydrate degradation profiling revealed potential cross-feeding interactions that may support metabolic cooperation among these taxa. *Catenibacterium mitsuokai* encodes enzymes for degrading trehalose, isomaltose, and dextrin into D-glucose. *Coprococcus* sp. was predicted to degrade β-glucans and converts maltose into trehalose, a substrate that can be used by *Catenibacterium mitsuokai.* Additionally, *Catenibacterium mitsuokai* may convert glycogen into glucose-1-phosphate, which can be further processed into D-glucose by *Phocaeicola dorei*. Together, these structured degradation pathways and substrate exchanges indicate a functionally integrated microbial cross-feeding network. This cooperative system may contribute to community stability under dietary pressure and may indirectly influence host metabolic outcomes through the modulation of tryptophan availability, redox regulation, and inflammation.

## Discussion

Despite growing evidence linking obesity and its management with gut microbiome alterations, comprehensive longitudinal studies integrating multi-omic datasets to observe clinical effects while also investigating underlying mechanisms remain scarce. To address this gap, our study investigated the effects of a structured one-year weight-loss intervention on anthropometric and clinical markers, gut barrier integrity, gut microbiota composition and function, and serum metabolomic profiles.

The nonsurgical one-year weight-loss intervention program led to a distinct reduction in body weight and body fat percentage. Moreover, it improved clinical markers and gut barrier function. Our findings revealed novel mechanistic insights by showing that particular microbial metabolites mediate the beneficial effects induced by the weight-loss program. Furthermore, we identified the serum level of diacylphosphatidylcholine C40:1 as a potential new biomarker able to predict the response of BMI to the intervention. Finally, we found a set of baseline parameters that predicted whether an individual would strongly benefit from the intervention. Our study provides new insights that might form the basis for novel precision nutrition approaches, allowing us to allocate individuals who are unlikely to benefit from this intervention to other weight-loss therapies.

Although the intervention led to marked improvements in anthropometric and clinical parameters during the initial 6 months, some of these effects were less pronounced at 12 months, including the reductions in BMI, body fat percentage, waist circumference, LDL cholesterol, and fecal zonulin. Nevertheless, these changes remained distinct and statistically significant compared with baseline. Notably, several parameters tended to show greater improvement at 12 months than at 6 months, including increased HDL cholesterol and fecal SCFA levels as well as decreased plasma LBP levels. This pattern reflects the well-documented challenge of weight-loss maintenance, in which initial improvements often plateau or partially regress over time [27]. The partial attenuation of several outcomes during the maintenance period may reflect both challenges in sustaining behavioral changes and persistent physiological adaptations to weight loss, including appetite-promoting hormonal changes and reductions in energy expenditure that favor weight regain [28]. Accordingly, weight maintenance may need to be treated as a distinct intervention phase, with continued individualized contact, self-monitoring and professional feedback, as well as renewed support for dietary and physical-activity adherence rather than simply continuing the initial intervention [29]. At the same time, our results suggest that certain benefits may emerge only after prolonged intervention. Our findings highlight the importance of long-term support strategies and raise critical questions about the durability of specific metabolic adaptations. Future interventions may benefit from identifying which early improvements are most strongly associated with sustained outcomes and from designing follow-up strategies that help maintain these benefits by directly supporting the underlying biological processes involved.

Recent meta-analyses and clinical studies suggest that weight loss is associated with changes in the composition of the gut microbiota [7, 21, 30–32]. Our findings extend these reports by identifying specific predictive microbiome and metabolome features that drive these changes. The intervention led to changes in the gut microbiota composition and increased fecal SCFA levels. For example, we reported an increase in the abundance of *Akkermansia*, a bacterial genus that has already been linked to improved metabolic health, reduced inflammation, and potential benefits for intestinal health and diseases such as obesity and diabetes [33, 34]. Notably, however, some studies have reported potentially harmful effects of these bacteria under specific conditions, such as a fiber-depleted diet [35]. It appears that associations between *Akkermansia* and weight loss are a consistent finding across energy-restricted diets, pharmacotherapy, and bariatric surgery [30, 36].

Our mediation analysis revealed a potential mechanistic link between the microbiome and gut barrier function through SCFAs. In particular, *Lachnospiraceae* was found to decrease plasma LBP levels; this effect was mediated primarily by butyrate and to a lesser extent by propionate. These findings are consistent with prior studies showing that SCFAs enhance gut barrier function by reducing epithelial permeability and limiting endotoxin translocation [37–40] and that the family *Lachnospiraceae* is known to produce SCFAs [41]. Furthermore, *Lachnospiraceae* may influence gut barrier function through bile-acid metabolism, as some members encode or exhibit bile-acid-modifying enzymes, including bile salt hydrolases and hydroxysteroid dehydrogenases, which can alter intestinal bile-acid pools [42, 43]. Because bile acids affect epithelial barrier integrity [44] and mucosal immune responses via receptors such as FXR and TGR5 [45, 46], this pathway may represent an additional mechanism linking *Lachnospiraceae* to gut barrier function.

Notably, LBP and zonulin, two established markers of intestinal barrier function [11], were associated with different predictors in our network analysis (Fig. 5B), reflecting their distinct biological roles. LBP is an acute-phase protein synthesized by the liver in response to bacterial lipopolysaccharide (LPS) translocating across the intestinal epithelial barrier into the systemic circulation. In our study, LBP levels were primarily associated with the gut microbiota-derived SCFAs propionate and butyrate. In contrast, zonulin is produced by intestinal epithelial cells and modulates intercellular tight junctions, thereby increasing paracellular permeability. In our network analysis, zonulin was primarily associated with *Peptococcaceae* but not with an SCFA. These differential associations are biologically plausible. Propionate and butyrate strengthen epithelial barrier function by increasing tight junction stability and lowering intestinal permeability [37–40]. Consequently, these SCFAs would be expected to primarily associate with systemic LBP levels, which rise only if LPS reaches the circulation. Compared to systemic LBP, zonulin may be more closely linked to particular bacterial taxa capable of directly interacting with epithelial cells, thereby triggering its release. While these hypotheses warrant further investigation, they support the notion that LBP and zonulin capture complementary, rather than interchangeable, aspects of intestinal barrier function [11, 13].

The results of our mediation analysis further revealed that *Bacteroides vulgatus* was associated with fasting blood glucose through the mediating variable of PC ae C44:6. Although studies investigating the clinical effects of PC ae C44:6 are limited and little is currently known about the physiological effects of this PC species, our results align with other studies linking the gut microbiota, phosphatidylcholine levels, and glucose homeostasis [47, 48]. In particular, a study in women with obesity revealed that inulin-type fructan supplementation decreased the abundance of *Bacteroides vulgatus* and altered serum phosphatidylcholine levels [48]. Furthermore, in individuals at risk for type 2 diabetes, serum levels of PC ae C44:6 were altered in response to glucose challenge tests, indicating that this metabolite is associated with an impaired glucose response [49].

A promising way to increase the success rates of weight-loss interventions is to identify individuals who are likely to benefit from such an intervention before they start the program. To do so, it is necessary to identify different patterns of response to the interventions or different phenotypes [50]. In our study, we deliberately chose not only to focus on body weight loss but also to consider the combined improvement across all clinical parameters assessed. This approach can classify participants as major responders based on broad clinical improvement, not solely weight loss. Our analyses revealed distinct differences in how the same intervention affected anthropometric and clinical parameters, the gut microbiota composition, and the serum metabolome. This is in line with previous studies, showing that diet homogeneity did not decrease interindividual variability in uremic microbiome-dependent metabolites [51]. Our findings of high interindividual variability are consistent with those of previous studies reporting substantial variability in weight-loss success following dietary and lifestyle interventions [52–57], highlighting the need for personalized intervention strategies, as identical approaches may not be equally effective for all individuals.

Among the prediction models, we found that models incorporating clinical and microbiome features and selected PCs demonstrated the strongest performance. Specifically, the resting metabolic rate, body fat percentage, leukocyte count, and PC aa C40:1 level were consistently identified as important across different models, highlighting their potential as predictive biomarkers. The predictive value of PC aa C40:1 was further supported by other analyses, namely, the dynamic Bayesian network and gradient boosting model, which identified this metabolite as a potential predictor of BMI loss. While the physiological effects of PC aa C40:1 are insufficiently understood, our findings align with prior metabolomic studies reporting that this PC is associated with BMI [58, 59]. To our knowledge, PC aa C40:1 has not been previously identified as a potential predictive marker for BMI response.

Additionally, the baseline levels of two bacteria, namely, *Phocaeicola dorei* and *Bacteroides vulgatus*, played a key role in predicting individual responses to the weight-loss intervention program. *Phocaeicola dorei* has been associated with improved metabolic health [60] and was also found to be a strong predictor of weight loss in another clinical trial [31]. The higher baseline abundance of *Phocaeicola dorei* in minor responders may reflect an altered immunometabolic state affecting responsiveness to the weight-loss intervention. In mice fed a high-fat diet, the administration of *Bacteroides vulgatus* attenuated body weight gain [61] and decreased body weight, insulin resistance, and intestinal permeability [62]. In humans, bariatric surgery increased the abundance of *Bacteroides vulgatus* [63]. Notably, while *Bacteroides vulgatus* has been linked to protease-driven mucus degradation and barrier disruption in severe inflammatory conditions like ulcerative colitis [64], we found no association between *B. vulgatus* and the barrier markers LBP and zonulin, suggesting different microbial dynamics in acute inflammatory states like ulcerative colitis and chronic metabolic conditions in obesity. In summary, the association between *Bacteroides vulgatus* and weight loss is not yet fully understood and requires further investigation. When considered alongside these findings, our results emphasize the potential of a multivariable prediction model as a novel tool in precision nutrition to combat obesity.

We have previously shown that after one year, 81% of participants receiving this intervention lost more than 10% of their initial body weight, with two-thirds of those achieving over 15% weight loss [3]. The program also reduced the prevalence of metabolic syndrome by 50% and lowered the frequency of hypertension from 47 to 29%, with these benefits persisting for up to three years [3]. Although most participants experienced substantial and sustained weight loss, 19% did not achieve sustained weight loss [3]. By identifying individuals unlikely to respond to nonsurgical, lifestyle-based interventions, our present analysis provides a foundation for personalized obesity treatment in the future. Specifically, our approach would identify individuals who are unlikely to benefit from this intervention and would allow us to allocate them to other weight-loss therapies, such as pharmacotherapy or bariatric surgery. This approach has the potential to improve overall program efficacy while sparing nonresponders the burden of ineffective, costly, and time-intensive interventions.

Although we observed improvements in several weight loss-related parameters, including CRP, gut barrier biomarkers, and fecal SCFA levels, these changes were not relevant predictors in our models and did not meaningfully contribute to the prediction of weight-loss success. This highlights the complexity and limitations of translating mechanistic insights primarily derived from preclinical models into clinically relevant outcomes in humans. Notably, the *Firmicutes/Bacteroidota* ratio, previously proposed as a hallmark microbial signature of obesity, remained unchanged throughout the intervention, which aligns with growing evidence challenging its relevance in human studies [65].

While our study provides valuable insights into the role of the gut microbiota and metabolomic markers in predicting weight loss outcomes, several limitations must be acknowledged. First, although all the participants followed the same formula-based diet during the first three months, they later transitioned to a “normal” balanced diet. Although all participants received the same guidance on the principles of this diet, individual dietary choices were not controlled or standardized and likely varied between participants. Although detailed dietary data were not collected throughout the one-year program, high compliance was observed. Since compliance did not differ between major and minor responders, we infer that their diet and lifestyle were comparable. Second, the intervention also included physical activity, mainly in the form of supervised group sport sessions. Physical activity was not recorded in the present study, which represents a limitation, as it may also influence clinical outcomes, serum metabolites, and the gut microbiota. However, we do not consider it likely that physical activity contributed significantly to the observed separation between major and minor responders or the prediction accuracy, because the compliance, including attendance at the sports sessions, showed similar participation between the groups. Third, although our sample size was sufficient for identifying key associations, both the small sample size and the selective study population of middle-aged individuals with obesity limit the generalizability of our findings to broader populations with different demographic and clinical characteristics. Future studies should aim to replicate these results in larger, more diverse cohorts, focusing on the key predictive parameters we identified in the present study. These larger cohorts would also enable external validation of the machine learning models we used in the present study to improve predictive accuracy. A limitation of our statistical pathway modeling is the assumption that microbiome features act exclusively as predictors and circulating clinical markers act solely as outcomes. In host physiology, this crosstalk can be bidirectional. For instance, systemic host metabolites can be secreted into or translocated across the gut lumen, directly altering the nutrient landscape and shifting microbial abundance. For example, host-derived lactate can be a substrate for *Veillonella* in the gut, which in turn produces propionate and enhances physical performance [66]. Future studies specifically designed to capture host-microbiome crosstalk should investigate this bidirectionality in more detail. Furthermore, the mediation analysis was restricted to candidate models in which the CLR-transformed microbial predictor was positively associated with the proposed metabolic mediator. This exploratory selection criterion was used to reduce the candidate model space in view of the cohort size and the high dimensionality of the integrated datasets and to focus on positive relative-abundance-metabolite pathways. It was not intended to imply that negative associations are necessarily artifactual or biologically unimportant. Consequently, the analysis may have overlooked inhibitory or depletion-associated pathways in which a reduction in the relative abundance of a microbial feature contributes to clinical improvement through a metabolic mediator. It may also have missed competing pathways in which direct and indirect effects operate in opposite directions. The identified mediation pathways should therefore be considered hypothesis-generating and not a comprehensive representation of all potential microbiome-metabolite-host relationships.

Our findings revealed novel mechanistic insights by showing that particular microbial metabolites mediate the beneficial effects induced by the weight-loss program. Furthermore, we identified baseline parameters allowing us to predict individual responses to a structured nonsurgical weight-loss intervention program. By combining these baseline parameters, one can identify individuals who are unlikely to benefit from this program and direct them toward alternative weight-loss strategies, such as pharmacotherapy or bariatric surgery. This approach has the potential to increase success rates in weight-loss therapy.

## Methods

### Weight-loss intervention program

All participants were part of an ongoing nonrandomized clinical study for individuals with obesity, with active recruitment taking place between 2009 and 2019 (ClinicalTrials.gov: NCT01344525). For this planned half-time interim analysis of primary and secondary outcomes, we included all participants who completed a one-year multidisciplinary weight-loss intervention (OPTIFAST^52^, Nestlé Health Science) between 2009 and 2019 and for whom anthropometric and clinical assessments, questionnaires, and biological samples were available for BL, 6 M, and 12 M. This resulted in a final sample of 50 individuals. All included blood and fecal samples underwent laboratory analysis in 2019 and 2020, and the resulting data were analyzed beginning in 2023. The study was approved by the ethics committee of the University Hospital of Tübingen, Germany (ID 87/2009BO1), and conducted at the obesity center located at the Metabolic Unit of the University of Hohenheim (Stuttgart, Germany). Patients or members of the public were not involved in the design, conduct, or reporting of this study.

The primary endpoint of the study was body weight loss, which we assessed as changes in BMI, body fat percentage, and waist circumference. The secondary endpoints included clinical assessments, gut microbiome analysis, targeted metabolomics, and gut barrier function, as well as additional endpoints that are described in detail in the study registration. The study was conducted in accordance with the Declaration of Helsinki. Written informed consent was obtained from all participants. The inclusion criteria were age between 18 and 65 years, a BMI of 30 kg/m^2^ or more and willingness to participate in the obesity treatment program. The exclusion criteria for the study were gastrointestinal disease; severe eating disorders; and treatment with anti-, pre-, or probiotics within three months before sample collection.

All participants underwent a defined and highly effective multidisciplinary weight-loss program described previously [3]. In brief, the program consisted of lifestyle modifications over 52 weeks on the basis of four modules: diet, exercise, behavioural therapy, and medical examinations. In the initial phase, meals were replaced by a low-calorie formula diet for approximately 3 months (ca. 850 kcal/day). This was followed by a transition phase in which the formula diet was gradually replaced by a balanced diet for approximately 3 months. Finally, the program progressed to a maintenance phase of approximately 6 months in which the participants followed a balanced diet, gradually increasing their calorie intake to a maximum of 2000 kcal/day. The study program comprised weekly group meetings at the study center. For the present analysis, we calculated the participants’ compliance, which was calculated as the percentage of weekly meeting attendance. The mean compliance of all 50 participants was 91% over the first 6 months and 83% over 12 months. The study design is shown in Fig. 1.

### Medical examinations

At all the study visits (BL, 6 M, 12 M), routine medical examinations were performed, comprising body weight and waist circumference measurements and a bioelectrical impedance analysis (BIA) to assess the phase angle and body fat percentage (device: Nutriguard MS, Data Input GmbH, Pöcking, Germany). At these visits, all participants were systematically asked by a physician about potential harms, side effects, or other adverse experiences related to the intervention. There were no severe events reported by the participants. Serum and plasma samples were collected in a fasting state by venipuncture. Fecal samples were collected on the day of the study visit or the day before and were immediately stored at −20 °C until they were transferred to −80 °C storage at the study center.

### Fecal and plasma biomarkers of gut barrier function and anti-inflammatory activity

Standard laboratory blood markers (cholesterol, blood glucose, CRP, etc.) were measured in a certified medical laboratory (Laborärzte Sindelfingen GbR, Sindelfingen, Germany). Zonulin and LBP (gut barrier biomarkers), as well as interleukin-10 (IL-10, an anti-inflammatory biomarker), were analyzed using enzyme-linked immunosorbent assays at the University of Hohenheim. Zonulin was assessed in fecal samples (REF: K5600; Immundiagnostik AG, Bensheim, Germany). In plasma, we assessed LBP (REF: KR6813; Immundiagnostik AG) and IL-10 (REF: DY217B-05; Bio-Techne GmbH, Wiesbaden, Germany) levels.

### Targeted serum metabolomics

We performed targeted serum metabolomics using the AbsoluteIDQ p180 Kit (Biocrates Life Sciences AG, Innsbruck, Austria), which quantifies 188 metabolites. Amino acid and biogenic amine profiles were determined by liquid chromatographic mass spectrometry; acylcarnitine, lysophosphatidylcholine, diacylphosphatidylcholine, acylphosphatidylcholine, sphingomyelin and hexoses were measured by flow injection mass spectrometry, as described in detail in the Supplementary Methods.

### Fecal DNA extraction and 16S rRNA sequencing

Bacterial DNA was extracted from the fecal samples, which had been diluted in a DNA stabilizer at the time of sample collection, using the PSP Spin Stool DNA Plus Kit (STRATEC Molecular GmbH, Berlin, Germany) following the manufacturer’s protocol. For gut microbiome assessment, 16S ribosomal RNA (rRNA) gene amplicons (2 × 300 bp) were sequenced on the MiSeq platform at the University of Minnesota Genomics Center (USA). The V5-V6 hypervariable regions of the 16S rRNA gene were amplified using the primers 784 F (5′-RGGATTAGATACCC-3′) and 1064R (5′-CGACRRCCATGCANCACCT-3′). We chose the V5-V6 region because it provides high resolution for *Bifidobacterium*, a genus that is highly associated with body weight [67], the main focus of our study. Using this region also ensured methodological continuity with earlier human intervention studies from our team in which we targeted the same region, including [21, 53, 67, 68]. To minimize batch effects, all 150 samples included in this study were sequenced in a single run. The raw sequencing data have been deposited in the Sequence Read Archive (SRA) of the NCBI (http://www.ncbi.nlm.nih.gov/sra) under BioProject identifier PRJNA1228582. Because of low sequence quality, 3 participants were excluded from all analyses including the gut microbiota data. An ASV table was created after chimera removal. The microbiome-related statistical analyses were conducted with centered-log ratio-transformed data to correct for compositionality [69] and were visualized with relative abundance data unless stated otherwise. The significance of diversity differences was tested using a linear mixed model controlling for individual variability, sex (female/male), and age group (two age groups, separated by the median age of 46 years), which were included as covariates. Beta diversity was assessed using Bray–Curtis distances computed via the vegan v2.6–2 package [70] and the Phyloseq R package [71] as well as weighted and generalized UniFrac (α = 0.5). Differences across samples were visualized using principal coordinate analysis (PCoA). Differences between time points at the phylum, genus, and ASV levels were investigated using linear models with the LinDA function from the MicrobiomeStat package [72]. For further details, see the Supplementary Methods.

### Quantification of fecal SCFA levels and fecal dry mass

As described in detail previously [73], the fecal samples were thawed, homogenized, diluted in water, and derivatized, after which SCFA levels were assessed by gas chromatography. To overcome bias due to different fecal water content, SCFA data were expressed in relation to each sample’s dry mass. Fecal dry mass was determined by weighing the fecal samples before and after oven drying.

### Statistical analyses and data handling

We categorized our data into three main datasets: 1) Clinical parameters (comprising anthropometric and laboratory measurements, gut barrier biomarkers, and fecal SCFAs, as shown in the Supplementary Excel File). Three subjects were removed from analyses of SCFA data because of numerous missing values. 2) Gut microbiota composition (comprising bacterial composition at the phylum, genus, and ASV levels). 3) Serum metabolomics (comprising a set of metabolites, assessed by targeted metabolomics). Here, metabolites whose levels were below the detection limit in more than 50% of the cohort were removed, resulting in 179 metabolites. To assess changes over time, we calculated the shift in each parameter between baseline and month 6 (6 M-BL) as well as between baseline and month 12 (12 M-BL).

### Ordination methods

Principal component analysis (PCA) was performed to provide an overview of the clinical and metabolomic datasets for the three time points. A linear mixed model was applied to identify single parameters that changed over time. Procrustes analysis was conducted to evaluate shifts among the baseline, 6-month, and 12-month time points in the three datasets. Euclidean distance was used to calculate the distance matrix for the shifts in the clinical and metabolomic datasets, whereas Aitchison distance was used for microbiome shift data. Associations between microbiome data and other metrics were visualized via PCoAs, with significance estimated using the protest function in the vegan package with 999 permutations.

### Sparse partial least squares regression and mediation analysis

Sparse partial least squares regression was applied to explore associations among microbiome, metabolomic, and clinical features. The identified key features were subsequently used to construct a correlation matrix, which was then visualized as a network diagram. From the network, we further proceeded to identify potential candidates for a subsequent mediation analysis. For details, see the Supplementary Methods.

### Dynamic Bayesian network and gradient boosting prediction

We applied Dynamic Bayesian network analysis to investigate how the baseline levels of each parameter from the clinical, microbiome and metabolome datasets may influence other parameters after the first 6 months of treatment. For details, see the Supplementary Methods.

### Clustering heatmap and random forest classification

We applied an unsupervised clustering approach to detect patterns of response based on the shift from baseline to month 6, including all parameters from the clinical dataset except SCFAs (because of missing SCFA data from 3 individuals). This analysis identified two distinct clusters: 24 major responders and 26 minor responders to the weight-loss program.

To identify the baseline features that were predictive of weight-loss response, we applied a random forest classification model. The models were trained on 70% of the total dataset of 50 individuals and tested on the remaining 30%. Multiple models were evaluated, including those based on individual feature sets—specifically, the microbiome, clinical features, diacylphosphatidylcholines, alkylacylphosphatidylcholines, and selected metabolites—and combined models integrating multiple data types (Supplementary Excel File). For details on the methods, see the Supplementary Methods.

### Elastic net regression analysis

Elastic net regression was used to identify microbial taxa associated with changes in selected host metabolites, including kynurenine, alpha-AAA, and putrescine [74]. Only metabolites with retained ASV predictors with non-zero elastic-net coefficients were carried forward into downstream microbial association and pathway analyses. The metabolic pathways linking microbial taxa, kynurenine metabolism, and starch/sugar degradation were manually annotated using reference data from MicrobiomeNet [75, 76] and the KEGG Pathway database [77, 78]. For details, see the Supplementary Methods.

### Statistical analysis and visualization

All the statistical analyses were conducted in RStudio 2024.09.0 using R version 4.4.1 (2024–06–14). Ordination methods (PCA, PCoA, and PLS-DA) were used to visualize the multi-omics data and explore overall patterns. Variations in the microbiome, clinical, and metabolomic datasets across different time points were assessed using PERMANOVA (adonis2 package in R), with significance tested using 999 permutations. A *p* value < 0.05 was considered statistically significant. For multiple hypothesis testing in linear models and correlation analyses, FDR correction was applied, and an adjusted p value (p_adj_) < 0.1 was considered statistically significant. Missing values were replaced by the medians of their respective time points. All plots were visualized using the ggplot2 package. Flow plots were visualized using the ggalluvial package [79], and all networks were visualized using the igraph package [80]. ROC curves from the prediction models were visualized using the pROC package [81]. All linear models and prediction models were controlled for sex and age group, with individual variation considered when time points were included.

## Supplementary Information

Supplementary Material 1: Supplementary Figures.

Supplementary Material 2: Supplementary Methods.

Supplementary Material 3: Supplementary Excel File.

## Data Availability

The 16S rRNA gene amplicon sequencing data were deposited in the NCBI Sequence Read Archive and are available for download under BioProject PRJNA1228582. Remaining de-identified individual participant data, methods, and study materials, as well as the study protocol and statistical analysis plan will be made available from the corresponding authors upon reasonable request.
