# Correction: Baseline gut microbiome and metabolome profiles predict weight loss after a structured lifestyle intervention

**DOI:** 10.1186/s40168-026-02597-1

**Published:** 2026-11-13

**Authors:** Benjamin Seethaler, Maryam Basrai, Nathalie M. Delzenne, Jens Walter, Nguyen K. Nguyen, Stephan C. Bischoff

**Affiliations:** 1 Institute of Nutritional Medicine, University of Hohenheim, Fruwirthstr. 12, Stuttgart, 70593 Germany; 2 Metabolism and Nutrition Research Group, Louvain Drug Research Institute (LDRI), UCLouvain, Université Catholique de Louvain, Brussels, Belgium; 3 APC Microbiome Ireland, Department of Medicine, and School of Microbiology, University College Cork, Cork, Ireland; 4 Department of Microbiology and Immunology, School of Biomedical Science, Faculty of Medicine and Health Sciences, McGill University, Montreal, QC Canada

**Correction: Microbiome 14, 207 (2026)**

**https://doi.org/10.1186/s40168-026-02501-x**

The original article contained an incomplete competing interests statement. The statement has been updated to read: ‘Stephan C. Bischoff is employed by the University of Hohenheim and also serves as Medical Director of the multimodal weight-reduction program described in this article, and he receives a salary for both roles.’”

The original article [1] has been updated.
