# Supplementary material for: Baseline gut microbiome and metabolome profiles predict weight loss after a structured lifestyle intervention
Source: Microbiome. 2026 Aug 28;14:207. doi: 10.1186/s40168-026-02501-x (PMC13525661; doi:10.1186/s40168-026-02501-x)
Supplement: Supplementary file 1 [file 40168_2026_2501_MOESM1_ESM.pdf]

Supplementary Figures from the publication:

Baseline gut microbiome and metabolome profiles predict weight loss after a structured lifestyle intervention, Seethaler *et al*.

- **Figure S1 (page 2)**
- **Figure S2 (page 3)**
- **Figure S3 (page 4)**
- **Figure S4 (page 5)**

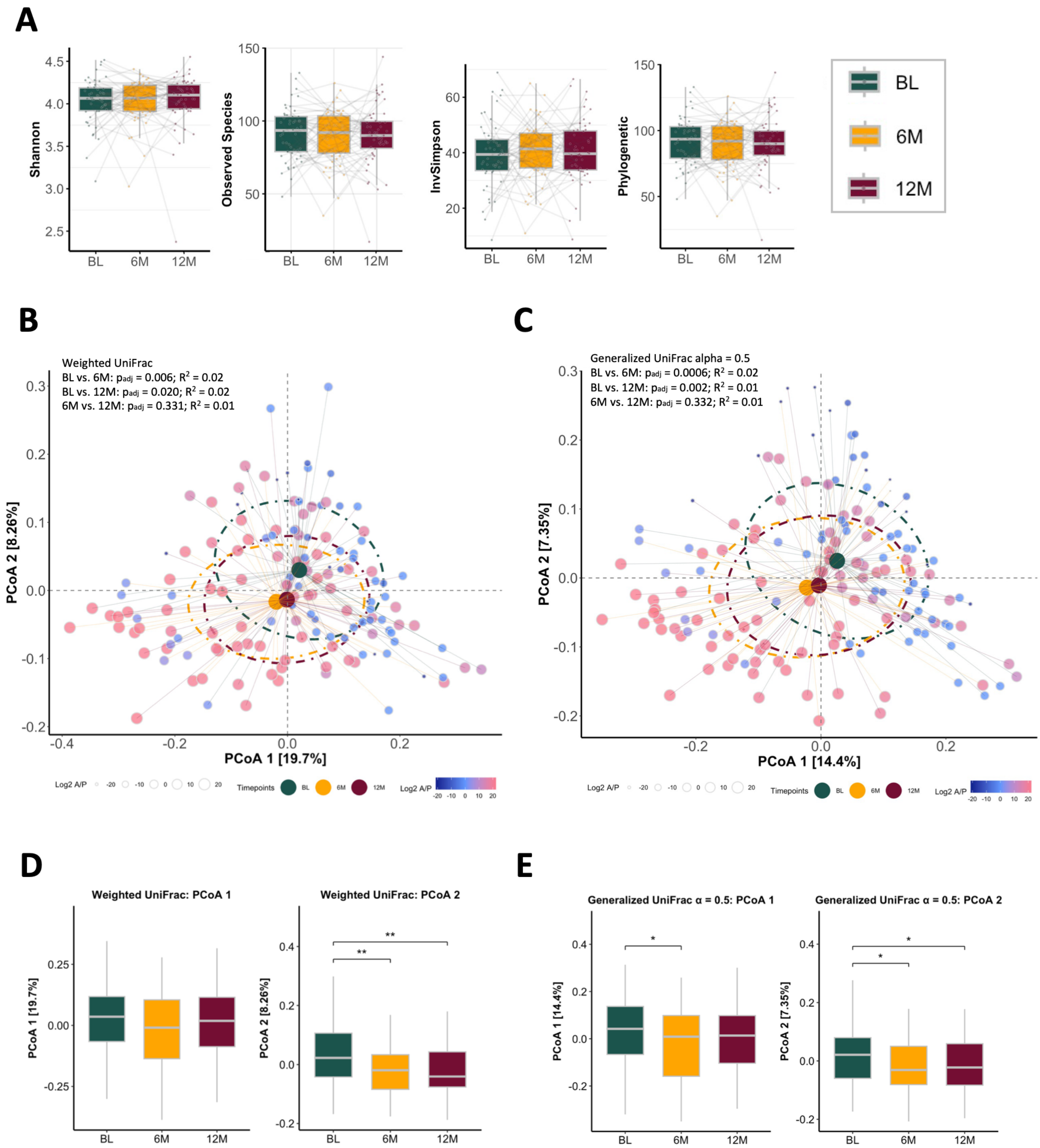

Figure S1. A, Using 16S rRNA sequencing, we found no effect of the intervention on gut microbial alpha diversity between baseline (BL) and month 6 (6M) as well as month 12 (12M) (all $p_{adj} > 0.1$, linear mixed models). **B,C,** Principal coordinate analysis (PCoA) plot depicting the beta diversity of the gut microbiome on the basis of weighted UniFrac (B) and generalized UniFrac (C) at baseline (BL), month 6 (6M), and month 12 (12M) (n = 141). An ordination analysis was performed using a permutational multivariate analysis of variance (PERMANOVA), adjusted for sex and individual variations. **D,E,** Boxplots showing PCoA1 and PCoA2 for each time point in detail (linear mixed models; *p < 0.05, **p < 0.01).

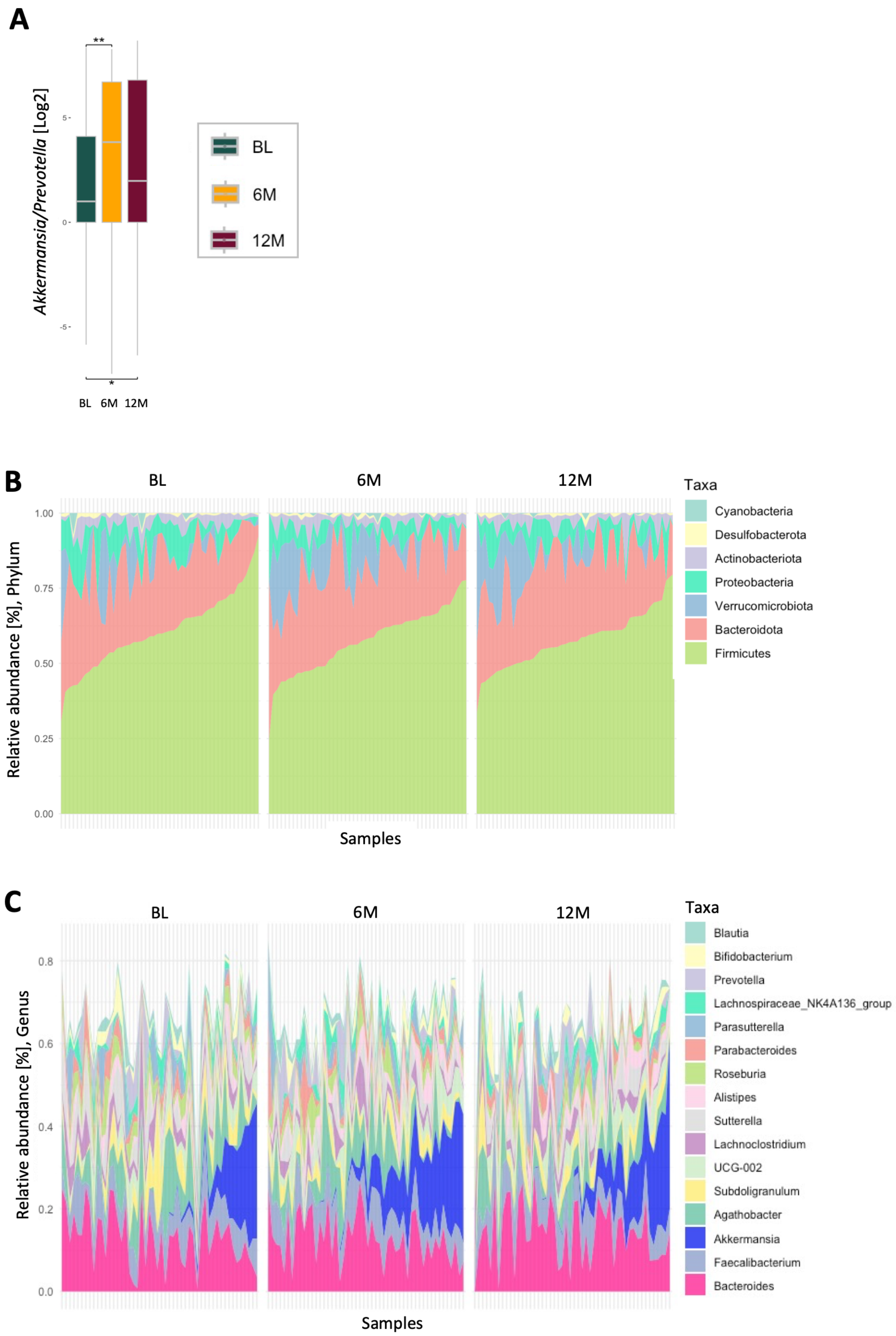

Figure S2. A, Boxplots showing the *Akkermansia/Prevotella* ratio at baseline (BL), month 6 (6M), and month 12 (12M) (linear mixed models; *p < 0.05, **p < 0.01). **B,** Relative abundance of the phyla. There was a higher relative abundance of the phylum Verrucomicrobiota at 6M (padj < 0.001) and 12M (padj = 0.009), as compared to BL. **C**, Relative abundance of bacterial genera at three time points: BL, 6M, and 12M (n = 141).

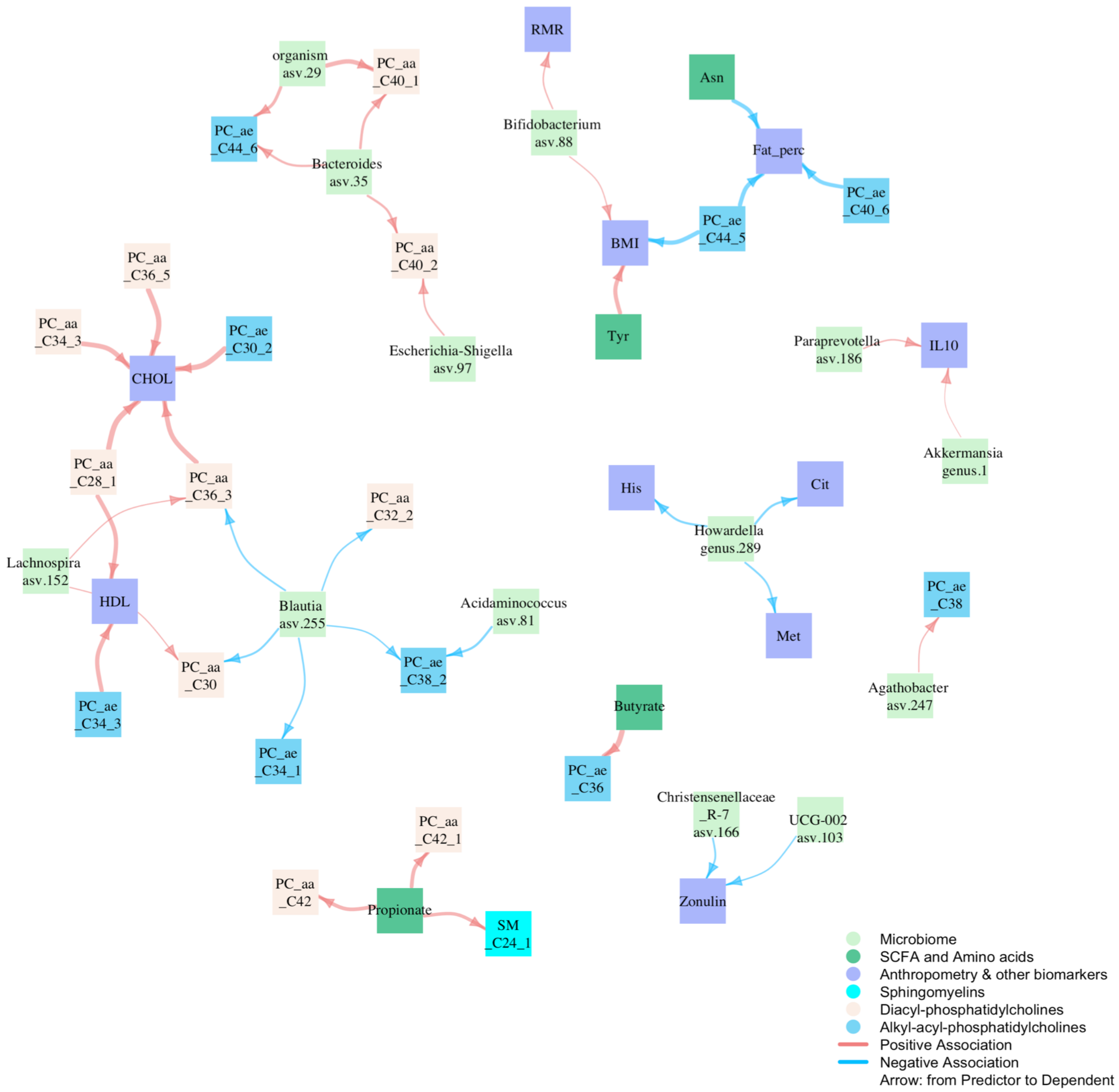

Figure S3. Network visualization of the significant associations among the changes in microbial taxa (CLR-transformed ASVs), metabolites, and clinical outcomes between baseline (BL) and month 12 (12M) (12M-BL) (n = 47 - 50). Key features were selected through sparse Partial Least Squares regression and subjected to Spearman correlation analysis. Only significant associations (p_{adj} < 0.1) were retained and then further adjusted for sex and age using linear models. All nodes are shown with a single shape regardless of data type and represent individual features (microbiome, metabolites, or clinical variables). Microbiome features exclusively act as predictors, while anthropometry and other biomarkers function solely as outcomes. Metabolites served as either predictors or outcomes depending on their role in the regression model. The colors of the circles indicate different parameter groups.

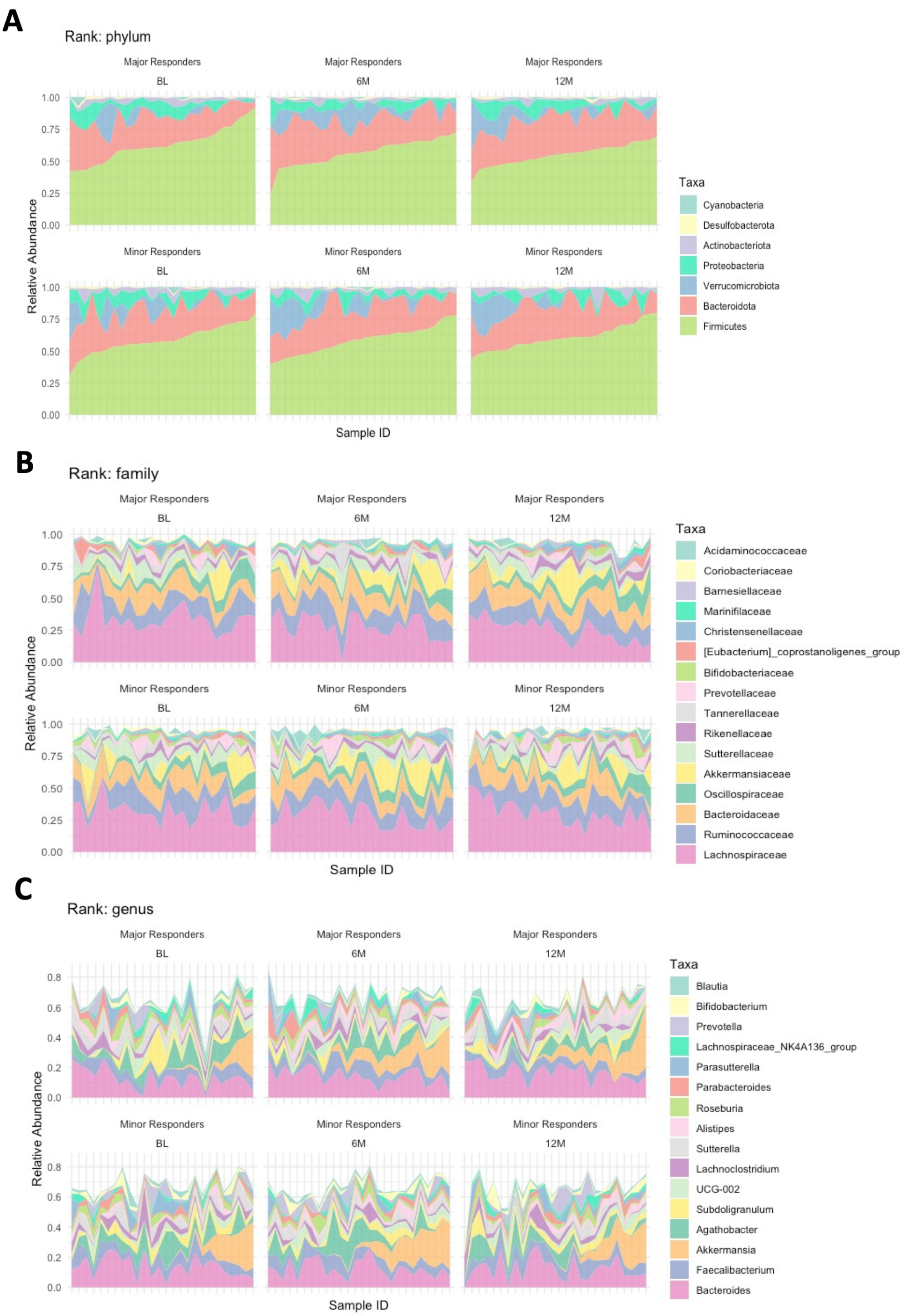

Figure S4. Relative abundance of bacterial taxa on different taxonomic levels (A, phylum; B, family; C, genus). Shown are the data for major and minor responders (see Figure 7) for baseline (BL), month 6 (6M), and month 12 (12M) (n = 141).
