# Supplementary material for: Baseline gut microbiome and metabolome profiles predict weight loss after a structured lifestyle intervention
Source: Microbiome. 2026 Aug 28;14:207. doi: 10.1186/s40168-026-02501-x (PMC13525661; doi:10.1186/s40168-026-02501-x)
Supplement: Supplementary file 2 [file 40168_2026_2501_MOESM2_ESM.pdf]

Baseline gut microbiome and metabolome profiles predict weight loss after a structured lifestyle intervention

Benjamin Seethaler, Maryam Basrai, Nathalie M. Delzenne, Jens Walter, Nguyen K. Nguyen, Stephan C. Bischoff

Supplementary Methods

1. *Targeted serum metabolomics*

Using the AbsoluteIDQ® p180 Kit (Biocrates Life Sciences AG, Innsbruck, Austria) we quantified 188 serum metabolites by following the manufacturer's manual. Amino acid and biogenic amine profiles were determined by liquid chromatographic mass spectrometry (LC-MS/MS); Acylcarnitine, lysophosphatidylcholine, diacyl-phosphatidylcholine, acyl-phosphatidylcholine, sphingomyelin and hexoses by flow injection mass spectrometric (FIA-MS/MS). The analyses were conducted on a SCIEX 5500 QTRAP mass spectrometer (SCIEX, Darmstadt, Germany) equipped with an Agilent 1290 UHPLC column (Agilent Technologies, INC., Santa Clara, CA, USA). Identification and quantification of metabolites were performed based on specific multiple reaction monitoring transitions and internal standards. After a pre-processing step (peak integration and concentration determination from calibration curves) using Analyst® 1.7 software (SCIEX), data were further processed in the Biocrates MetIDQ software. Concentrations of metabolites assessed by FIA were directly calculated in MetIDQ. LC-MS/MS, FIA-MS/MS, and peak integration in the Analyst® software were performed at the Core Facility Hohenheim (mass spectrometry unit). As a last step, the data were normalized using the Biocrates MetIDQ software.

2. *Fecal DNA Extraction and 16S rRNA Sequencing*

The raw sequencing data in the FASTQ format were processed using QIIME 2 (v2021.11, https://qiime2.org/). The sequences were demultiplexed following the public tutorial for the

Casava 1.8 paired-end demultiplexed format. Quality control and denoising were performed using the DADA2 algorithm [1] via the qiime dada2 denoise-paired command, generating an amplicon sequence variant (ASV) table after chimera removal.

Taxonomic classification of ASVs was performed using the QIIME 2 feature-classifier plugin [2] with the classify-sklearn function. Taxonomy was assigned against the pre-trained SILVA v138 database [3] specific to the V5-V6 region using the RESCRIPt pipeline [4]. ASVs corresponding to untargeted sequences and singletons were removed. Extremely low-abundant ASVs, defined as those appearing in fewer than three counts per sample and present in less than 10% of all samples, were also excluded.

Following quality control, sequencing depth was inspected before diversity analysis. The raw ASV table contained 2,620,560 reads across 150 samples, with a mean sequencing depth of 17,470 ± 4,100 reads per sample median: 17,449; range: 8,285–27,210. After removing non-bacterial and unassigned features, 2,578,907 reads remained, with a mean depth of 17,193 ± 4,195 reads per sample. After prevalence filtering feature count >3 reads in >10% of samples, the final diversity-analysis table contained 1,752,002 reads, 341 ASVs, and 150 samples, with an average of 11,680 ± 3,392 reads per sample median: 11,546; range: 4,988–21,083. All retained samples exceeded the minimum sequencing-depth threshold of 2,000 reads. Sequencing depth was also comparable across time points. In the final filtered ASV table, mean sequencing depth was 12,090 ± 3,340 reads at baseline, 11,533 ± 3,308 reads at 6 months, and 11,418 ± 3,554 reads at 12 months. Therefore, no time point-specific low-depth subset was identified.

The microbiome-related statistical analyses were conducted using centered-log ratio-transformed data (CLR) to correct for compositionality [5] and were visualized using relative abundance data unless stated otherwise.

Alpha diversity was estimated using the Shannon index, Inverse Simpson index, Observed Species, and Phylogenetic Index (Faith's phylogenetic diversity), on the raw count amplicon

sequence variants (ASVs) table after filtering out contaminants using the Phyloseq R package [6]. The significance of diversity differences was tested using a linear mixed model controlling for individual variability, sex (female/male), and age group (two age groups, separated by the median) included as covariates. Beta diversity was assessed using Bray-Curtis distances computed via the vegan v2.6-2 package [7] and the Phyloseq R package [6]. To address phylogeny-aware community structure, representative ASV sequences were aligned using DECIPHER [8], and a maximum-likelihood ASV phylogenetic tree was inferred using phangorn [9]. This tree was subsequently used to compute weighted UniFrac distances with Phyloseq and generalized UniFrac distances with the GUniFrac package using $\alpha = 0.5$. Differences across samples were visualized using Principal Coordinate Analysis (PCoA) ordination. Differences between the time points at the phylum, genus, and ASV levels were investigated using linear models with the LinDA function from the MicrobiomeStat package [10].

3. *Statistical analyses and data handling*

We categorized our data into three main datasets: **1) clinical parameters** (comprising anthropometric and laboratory measurements, gut barrier biomarkers, and fecal short-chain fatty acids [SCFAs]), as shown in the Supplementary Excel File. Three subjects were removed from analyses comprising SCFA data due to extensive missing values. **2) Gut microbiota composition** (comprising bacterial composition on phylum, genus, and ASV levels). Here, due to low sequencing quality, we excluded 3 participants. Therefore, all analyses including gut microbiota data are performed with 47 participants. **3) Serum metabolomics**, assessed by targeted metabolomics. Metabolites with levels below the detection limit in more than 50% of the cohort were removed, resulting in 179 of 188 metabolites being included in the analysis.

Sparse Partial Least Squares and Mediation Analysis

Sparse Partial Least Squares regression was applied to explore associations among microbiome, metabolomic, and clinical features using the mixOmics package [11]. This approach identified single key features that contributed to the relationships found between the main datasets in the Procrustes analysis. These identified key features were subsequently

used to construct a correlation matrix using Spearman's correlations. Here, only significant correlations with a False Discovery Rate (FDR) < 0.1 were further assessed using a linear model, adjusted for sex and age. The final set of significant relationships (FDR < 0.1) was visualized as a network diagram.

Based on the Procrustes analysis, which demonstrated a significant association between microbiome shifts and other measured features, microbiome-related nodes function as primary predictors in the networks, while clinical features served as dependent variables. Features from the metabolomic dataset served as either a predictor or a dependent variable. If a metabolomic feature was linked to a microbiome feature, it was defined as a dependent variable, whereas if it was linked to a clinical feature, it was categorized as a predictor.

From the network, we further proceeded to identify potential candidates for a subsequent mediation analysis based on the following conditions: (i.) direction: predictor (microbiome dataset) → mediators (metabolomics dataset) → clinical outcome (clinical dataset); (ii.) Association: predictors are expected to have a positive association with the mediator. This selection led to ten mediation models for the baseline (BL) to 6-month (6M) shifts (6M-BL) and one model for the 12-month shift (12M-BL) which were tested. Mediation analysis was conducted using the mediation R package with 500 bootstrap replications. Only models in which both the mediated (indirect) and total effects were significant (FDR < 0.1) were considered as meaningful.

Dynamic Bayesian Network and gradient boosting prediction

We applied Dynamic Bayesian Network analysis using the bnlearn package [12]. We aimed to investigate how baseline levels of each parameter from the clinical, microbiome and metabolome datasets may influence other parameters after the first 6 months of treatment. Variables with high collinearity (Pearson's R > 0.85) were excluded for correct interpretability. The Hill-Climbing algorithm was used to learn the network structure with a maxp parameter of 10, optimizing the Bayesian Information Criterion. The dataset contained 641 features, including 17 at 6M, resulting in 10,608 possible associations restricted to BL features

predicting 6M outcomes. A blacklist ensured that only BL features influenced 6M outcomes. The structure was fitted using bn.fit, estimating conditional probability distributions. The final network, filtered using a residual threshold of 1.5, showed 28 connections for visualization.

To further validate the observed key relationships, we applied gradient boosting regression using the xgboost package [13]. Here, SCFA data were included which is why the total sample size was 47. The model was trained on 70% of the 47 subjects and focused on predicting a subset of 6-month outcomes that were primarily influenced by the baseline values of other variables, rather than by their own baseline values. Sex and age group were also included as covariates in the model. Performance was evaluated using Root Mean Squared Error, Mean Absolute Error, and R^2. Among multiple predictive models tested, the final model was selected based on achieving a high R^2 (> 0.15) and a low Mean Absolute Error (< 0.5), ensuring both strong explanatory power and minimal prediction error. These criteria were chosen as the data were scaled, making Mean Absolute Error an appropriate measure for assessing prediction accuracy. Finally, based on these selection criteria only two predictive models were considered and interpreted, ensuring robust and reliable insights into the key relationships.

Clustering Heatmap and Random Forest Classification

We applied an unsupervised clustering approach using the hierarchical clustering method (hclust) with the "ward.D" method to detect patterns of response based on the shift from baseline to month 6 (6M-BL) including all parameters from the clinical dataset except SCFAs (excluded due to missing data from three individuals) in all 50 participants. This analysis identified two distinct clusters: 24 major responders and 26 minor responders to the weight-loss program. The features which contributed to the differentiation of the two types of responders were further identified using a linear model, controlled for sex and age, assessing shifts in features from both 6M-BL (Figure 7B) and 12M-BL (Figure 7C).

To identify the baseline features which were predictive of weight-loss response, we applied a random forest classification model using the ranger package [14]. To avoid overadjustment,

we included the clinical features and metabolomic variables which were found to be relevant by the Dynamic Bayesian Network (Supplementary Excel File). Additionally, we included all diacyl-phosphatidylcholines and alkyl-acyl-phosphatidylcholines that were featured in the network identified by Sparse Partial Least Squares regression, as they contributed prominently to the metabolic response. For microbiome features, we focused on the *Bacteroides* genus, including all ASVs closely related to *Phocaeicola dorei*, *Bacteroides vulgatus*, and *Bacteroides uniformis*, based on their well-documented roles in weight loss and obesity outcomes [15,16]. In the random forest classification, features showing high pairwise correlation ($r > 0.7$) were excluded to reduce multicollinearity, resulting in a final set of 31 baseline features, including sex and age. Model performance was evaluated using random forest classification with 10-fold cross-validation and recursive feature elimination. By iteratively removing the least important features based on random forest variable importance, recursive feature elimination helped to optimize the final model and reduce noise from redundant variables. The models were trained on 70% of the total dataset of 50 individuals and were tested on the remaining 30%. Multiple models were evaluated, including those based on individual feature sets-specifically microbiome, clinical features, diacyl-phosphatidylcholines, alkyl-acyl-phosphatidylcholines, and selected metabolites and combined models integrating multiple data types (Supplementary Excel File). Important features were extracted from each cross-validation fold and visualized based on their average Gini importance. Performance metrics included accuracy and area under the ROC curve (AUC) which were calculated using the testing dataset by the caret function. To further explore the separation between major and minor responders, a partial least squares discriminant analysis (PLS-DA) from mixOmics package, was performed using the top-ranked features identified from the model. Permutational multivariate analysis of variance (PERMANOVA) was conducted to statistically assess the degree of separation between the two groups.

Elastic Net Regression Analysis

Elastic net regression was used to identify microbial taxa associated with changes in selected host metabolites, including kynurenine, alpha-aminoadipic acid, and putrescine. Each of the three metabolites was modeled separately as an outcome variable. Only metabolites with retained ASV predictors with non-zero elastic-net coefficients were carried forward into downstream microbial association and pathway analyses. The predictor matrix consisted of 341 CLR-transformed ASV shifts (6M-BL). For each model, the cv.glmnet() function from the glmnet R package [17] was used to fit the Elastic Net with an alpha value of 0.5, which balances variable selection with the ability to retain correlated features. Coefficients were extracted using the coef() function at the lambda value corresponding to the minimum cross-validated error (lambda.min). ASVs with non-zero coefficients (excluding the intercept) were considered relevant predictors. Because the glmnet method does not compute p-values, results were interpreted based on the magnitude and presence of non-zero coefficients.

Finally, we investigated potential interactions and co-dependencies between bacterial genera. Following feature selection by Elastic Net, pairwise Spearman correlations were calculated between the selected ASVs. Only correlations with a FDR < 0.05 were considered significant and visualized in the microbial network. To confirm the strong associations among taxa identified in the network, microbial complementarity scores were calculated for each pair using genome-scale metabolic models via MicrobiomeNet [18,19]. Only ASVs assigned at the genus level were included in this analysis. Results were visualized in a directional flow chart, where bar width represents the level of predicted metabolic support from donor to receiver taxa. Finally, the metabolic pathways linking microbial taxa, kynurenine metabolism, and starch/sugar degradation were manually annotated using reference data from the MicrobiomeNet and the KEGG Pathway database.

References

1. Callahan BJ, McMurdie PJ, Rosen MJ, Han AW, Johnson AJA, Holmes SP. DADA2: High-resolution sample inference from Illumina amplicon data. Nat Methods. 2016;13:581–3. https://doi.org/10.1038/nmeth.3869

2. Bokulich NA, Kaehler BD, Rideout JR, Dillon M, Bolyen E, Knight R, et al. Optimizing taxonomic classification of marker-gene amplicon sequences with QIIME 2's q2-feature-classifier plugin. Microbiome. 2018;6:90. https://doi.org/10.1186/s40168-018-0470-z

3. Quast C, Pruesse E, Yilmaz P, Gerken J, Schweer T, Yarza P, et al. The SILVA ribosomal RNA gene database project: improved data processing and web-based tools. Nucleic Acids Res. 2013;41:D590–6. https://doi.org/10.1093/nar/gks1219

4. Robeson MS, O'Rourke DR, Kaehler BD, Ziemski M, Dillon MR, Foster JT, et al. RESCRIPt: Reproducible sequence taxonomy reference database management. PLoS Comput Biol. 2021;17:e1009581. https://doi.org/10.1371/journal.pcbi.1009581

5. Aitchison J. The Statistical Analysis of Compositional Data. Journal of the Royal Statistical Society Series B (Methodological). [Royal Statistical Society, Wiley]; 1982;44:139–77.

6. McMurdie PJ, Holmes S. phyloseq: an R package for reproducible interactive analysis and graphics of microbiome census data. PLoS One. 2013;8:e61217. https://doi.org/10.1371/journal.pone.0061217

7. Oksanen J, Blanchet FG, Friendly M, Kindt R, Legendre P, McGlinn D, et al. vegan: Community Ecology Package. R package version 25-7. 2020;

8. Wright E. Using DECIPHER v2.0 to Analyze Big Biological Sequence Data in R. The R Journal. 2016;8. https://doi.org/10.32614/RJ-2016-025

9. Schliep KP. phangorn: phylogenetic analysis in R. Bioinformatics. 2011;27:592–3. https://doi.org/10.1093/bioinformatics/btq706

10. Zhou H, He K, Chen J, Zhang X. LinDA: linear models for differential abundance analysis of microbiome compositional data. Genome Biol. 2022;23:95. https://doi.org/10.1186/s13059-022-02655-5

11. Rohart F, Gautier B, Singh A, Lê Cao K-A. mixOmics: An R package for 'omics feature selection and multiple data integration. PLoS Comput Biol. 2017;13:e1005752. https://doi.org/10.1371/journal.pcbi.1005752

12. Scutari M. Learning Bayesian Networks with the bnlearn R Package. Journal of Statistical Software. 2010;35:1–22. https://doi.org/10.18637/jss.v035.i03

13. Chen T, He T, Benesty M, Khotilovich V, Tang Y, Cho H, et al. xgboost: Extreme Gradient Boosting. 2025 [cited 2025 Apr 22]; https://cran.r-project.org/web/packages/xgboost/index.html. Accessed 22 Apr 2025

14. Wright MN, Wager S, Probst P. ranger: A Fast Implementation of Random Forests [Internet]. 2024 [cited 2025 Apr 23]. https://cran.r-project.org/web/packages/ranger/index.html. Accessed 23 Apr 2025

15. Romaní-Pérez M, López-Almela I, Bullich-Vilarrubias C, Evtoski Z, Benítez-Páez A, Sanz Y. Bacteroides uniformis CECT 7771 requires adaptive immunity to improve glucose

tolerance but not to prevent body weight gain in diet-induced obese mice. Microbiome. 2024;12:103. https://doi.org/10.1186/s40168-024-01810-3

16. Jie Z, Yu X, Liu Y, Sun L, Chen P, Ding Q, et al. The Baseline Gut Microbiota Directs Dieting-Induced Weight Loss Trajectories. Gastroenterology. 2021;160:2029-2042.e16. https://doi.org/10.1053/j.gastro.2021.01.029

17. Friedman J, Hastie T, Tibshirani R. Regularization Paths for Generalized Linear Models via Coordinate Descent. J Stat Softw. 2010;33:1–22.

18. Lu Y, Hui F, Zhou G, Xia J. MicrobiomeNet: exploring microbial associations and metabolic profiles for mechanistic insights. Nucleic Acids Research. 2025;53:D789–96. https://doi.org/10.1093/nar/gkae944

19. Lu Y, Nguyen KN, Xia J. Interpreting Microbiome Signatures with MicrobiomeNet. Curr Protoc. 2026;6:e70338. https://doi.org/10.1002/cpz1.70338
